# The biosynthetic implications of late-stage condensation domain selectivity during glycopeptide antibiotic biosynthesis†
†Electronic supplementary information (ESI) available. See DOI: 10.1039/c8sc03530j


**DOI:** 10.1039/c8sc03530j

**Published:** 2018-10-10

**Authors:** Melanie Schoppet, Madeleine Peschke, Anja Kirchberg, Vincent Wiebach, Roderich D. Süssmuth, Evi Stegmann, Max J. Cryle

**Affiliations:** a The Monash Biomedicine Discovery Institute , Department of Biochemistry and Molecular Biology , EMBL Australia , Monash University , Clayton , Victoria 3800 , Australia . Email: max.cryle@monash.edu; b Department of Biomolecular Mechanisms , Max Planck Institute for Medical Research , Jahnstrasse 29, 69120 Heidelberg , Germany; c ARC Centre of Excellence in Advanced Molecular Imaging , Monash University , Clayton , Victoria 3800 , Australia; d Institut für Chemie , Technische Universität Berlin , Strasse des 17. Juni 124 , 10623 Berlin , Germany; e Interfaculty Institute of Microbiology and Infection Medicine Tübingen , Microbiology/Biotechnology , University of Tübingen , Auf der Morgenstelle 28, 72076 Tübingen , Germany . Email: evi.stegmann@biotech.uni-tuebingen.de; f German Centre for Infection Research (DZIF) , Partner Site Tübingen, Tübingen , Germany

## Abstract

Non-ribosomal peptide synthesis is a highly important biosynthetic pathway for the formation of many secondary metabolites of medical relevance. Due to the challenges associated with the chemical synthesis of many of the products of these assembly lines, understanding the activity and selectivity of non-ribosomal peptide synthetase (NRPS) machineries is an essential step towards the redesign of such machineries to produce new bioactive peptides. Whilst the selectivity of the adenylation domains responsible for amino acid activation during NRPS synthesis has been widely studied, the selectivity of the essential peptide bond forming domains – known as condensation domains – is not well understood. Here, we present the results of a combination of *in vitro* and *in vivo* investigations into the final condensation domain from the NRPS machinery that produces the glycopeptide antibiotics (GPAs). Our results show that this condensation domain is tolerant for a range of peptide substrates and even those with unnatural stereochemistry of the peptide C-terminus, which is in contrast to the widely ascribed role of these domains as a stereochemical gatekeeper during NRPS synthesis. Furthermore, we show that this condensation domain has a significant preference for linear peptide substrates over crosslinked peptides, which indicates that the GPA crosslinking cascade targets the heptapeptide bound to the final module of the NRPS machinery and reinforces the role of the unique GPA X-domain in this process. Finally, we demonstrate that the peptide bond forming activity of this condensation domain is coupled to the rate of amino acid activation performed by the subsequent adenylation domain. This is a significant result with implications for NRPS redesign, as it indicates that the rate of amino acid activation of modified adenylation domains must be maintained to prevent unwanted peptide hydrolysis from the NRPS due to a loss of the productive coupling of amino acid selection and peptide bond formation. Taken together, our results indicate that assessing condensation domain activity is a vital step in not only understanding the biosynthetic logic and timing of NRPS-mediated peptide assembly, but also the rules which redesign efforts must obey in order to successfully produce functional, modified NRPS assembly lines.

## Abstract 2

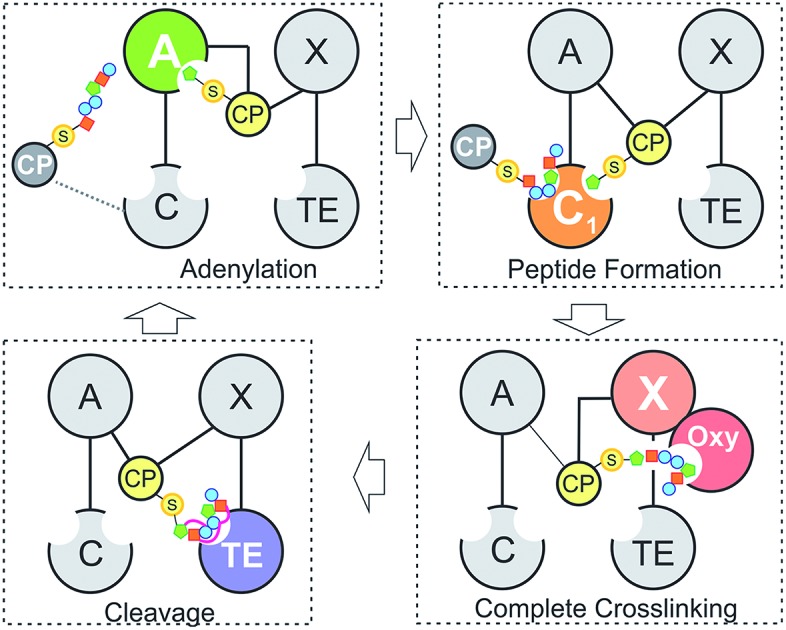
The condensation domain synthesising the last peptide bond in glycopeptide antibiotic biosynthesis has a preference for linear peptide substrates, with effective peptide formation linked to the rate of amino acid activation by the preceding adenylation domain.

## Introduction

**Fig. 1:**
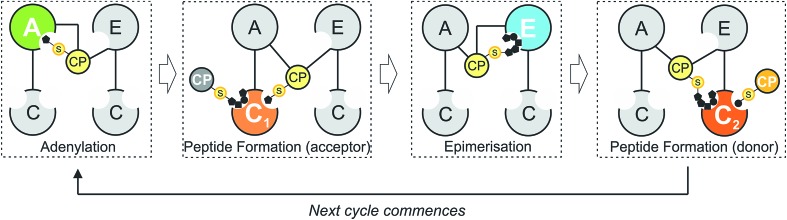
Schematic representation of peptide bond formation as performed by a NRPS extension module containing an epimerisation domain. Amino acid selection and subsequent activation are performed by the adenylation (A)-domain (first panel, starting from the left), after which the amino acid is transferred onto the PPant moiety of the neighbouring carrier protein (CP) domain. Following this, the aminoacyl-CP then acts as the acceptor in peptide bond formation performed by the upstream condensation domain (second panel). At this point, the peptide present on the upstream donor CP is transferred onto the acceptor aminoacyl-CP, extending the peptide by one residue. Depending on the stereochemistry required at the C-terminal position of the peptide, an epimerisation domain can alter the standard l-configuration into the non-natural d-form (third panel). The stereochemical state of the peptide is then assessed by the downstream C-domain, where the peptidyl-CP now acts as the donor substrate for the next round of peptide bond formation (fourth panel).

Natural products biosynthesis contains many examples of complex, bioactive molecules produced by the actions of equally complex enzymatic assembly lines. In particular, polyketide synthase (PKS) and non-ribosomal peptide synthetase (NRPS) assembly lines serve as potent examples of nature's ability to produce a diverse range of structures based on the assembly of repeating building blocks (acetate/malonate and amino acids, respectively).^1^–^4^ What makes both systems of great interest – in addition to the large number of important compounds produced by these pathways – is that such assembly lines typically consist of repeating groups of conserved catalytic domains clustered into modules, each responsible for the incorporation (and modification) of monomers into the growing product. In NRPS-mediated biosynthesis, a modular architecture allows the formation of peptides with greatly diversified amino acid content, modifications and altered stereochemistry to that typically seen from peptides derived from ribosomal synthesis.^3^,^5^ Central to NRPS synthesis are three domains: adenylation (A)-, peptidyl carrier protein (PCP)- and condensation (C)-domains, which together form a minimal unit required to extend a growing non-ribosomal peptide by one amino acid residue (Fig. 1).^3^ Selection and activation (adenylation) of the desired monomer is performed by the A-domain in an ATP-dependant process, which results in the initial activation of the desired monomer as an AMP adenylate.^6^ This highly activated monomer is then transferred onto the terminal thiol group of the phosphopantetheine arm of the adjacent PCP domain, resulting in the formation of a thioester bound aminoacyl-PCP species.^7^ Peptide bond formation is then performed in the C-domain, where two (typically) PCP-bound substrates are condensed such that the upstream “donor” amino acid/peptide is transferred onto the downstream “acceptor” aminoacyl-PCP, resulting in peptide bond formation and elongation of the peptide by one residue.^8^,^9^

Minimal NRPS modules are often supplemented by additional modification domains, arguably the most important of which are epimerisation (E)-domains.^3^ These domains are responsible for the epimerisation of the C-terminal residue of the PCP-bound peptide from the l to the d form, and are believed to act together with C-domains to ensure that the correct stereochemistry is maintained during NRPS-mediated synthesis (Fig. 1). Upon completion of the peptide chain, the peptide is removed from the NRPS, typically through the actions of a terminal thioesterase (TE) domain, which serves to act as a further point for structural diversification of the peptide.^10^ Given that the products of many NRPS assembly lines have important roles in medicine and that their structural complexity can limit their chemical synthesis at scale, the modular architecture of an NRPS is naturally highly attractive for potential redesign efforts to produce new bioactive peptide products.^4^ Such efforts are often restricted, however, due to our limitations in understanding the exact structure, selectivity and rate of these complex molecular machines: this makes understanding the fundamental process that underpin NRPS activity of key importance and crucial to the success of future enzymatic redesign efforts for these important systems.

Within non-ribosomal peptide synthesis, condensation (C)-domains play the essential role of catalysing amide bond formation between neighbouring PCP-bound substrates (Fig. 1).^9^,^11^ Whilst previously seen as little more than stereochemical gatekeepers during NRPS-mediated peptide synthesis – a role that they share with structurally related E-domains – C-domains have now been shown to perform highly diverse roles during NRPS biosynthesis. Examples include the formation of beta-lactam rings, multiple-step heterocyclisation reactions, peptide cyclisation, ester bond formation and complex transformations to produce modified amino acid residues.^3^,^8^,^12^–^16^ Beyond this expansion of conventional C-domain activity, many questions still remain concerning the specificity of C-domains during peptide bond formation, including selectivity for their upstream PCP-bound peptide substrates, the influence of *trans*-acting enzymes and the importance of coupling A-domain amino acid selection with the rate of C-domain activity. As *in vivo* studies have already demonstrated the potential for C-domains to display selectivity towards their peptide substrates,^17^,^18^ this makes a detailed characterisation of C-domain behaviour *in vitro* all the more pressing in order to understand the mechanism behind the apparent selectivity observed for these key NRPS domains.

**Fig. 2:**
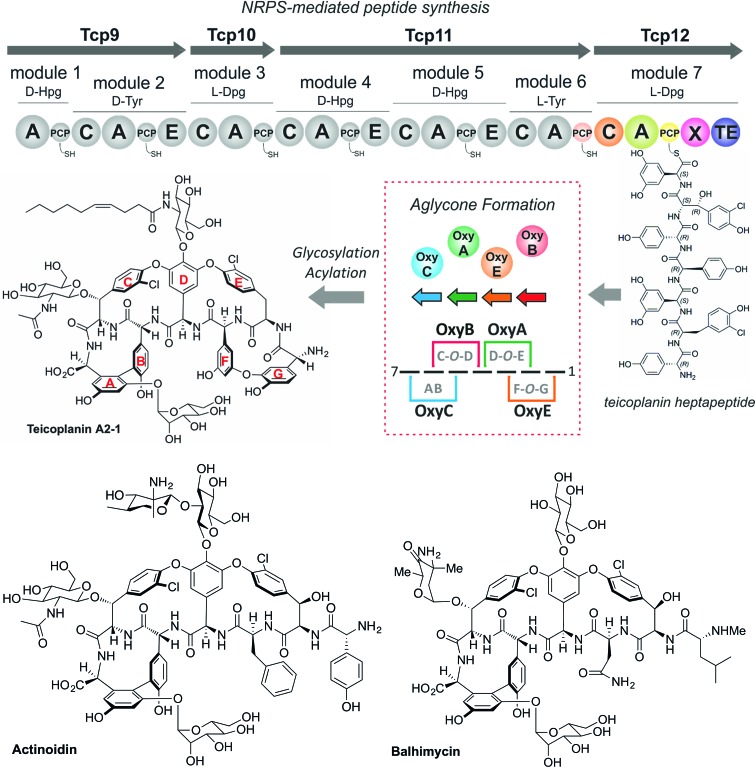
Biosynthetic scheme for the glycopeptide antibiotics (GPAs), exemplified for teicoplanin (type-IV GPA, upper panel) as well as related GPA structures relevant for this work actinoidin (type-II GPA, lower left) and balhimycin (type-I GPA lower right). Type-III GPAs possess the same core peptide sequence as type-IV GPAs. In GPA biosynthesis, the NRPS-mediated synthesis of a linear heptapeptide precursor is followed by an oxidative peptide cyclisation cascade of cytochrome P450 (Oxy) enzymes, which transform the linear peptide into its rigid, active form whilst the peptide remains bound to the NRPS machinery. In the biosynthesis of the three GPAs indicated here, the NRPS machinery remains the same from a domain and module perspective: the main differences between these GPA biosynthetic machineries are the number of Oxy enzymes and hence crosslinks installed in the cyclic peptide (3 – balhimycin/actinoidin; or 4 – teicoplanin), the presence of 3 (balhimycin) or 4 (actinoidin/teicoplanin) NRPS-encoding proteins, and the residues contained within the peptide that are dictated by the selectivity of the A-domains.

The glycopeptide antibiotics (GPAs) serve as a potent example of the need to study and understand non-ribosomal peptide synthesis: these heptapeptide natural products remain one of the last clinical antibiotics with activity against methicillin-resistant *Staphylococuus aureus* (MRSA).^19^ Their complex chemical structures and resulting difficulties in total synthesis are the reason that we remain reliant upon the natural biosynthetic pathways that produce these compounds for their clinical use (Fig. 2).^20^ GPAs rely on the interplay between a linear NRPS and a complex, late stage peptide cyclisation cascade comprising 3 or 4 cytochrome P450 monooxygenase enzymes (known as Oxy enzymes) (Fig. 2).^21^,^22^ It is known that the cyclisation cascade in GPA biosynthesis occurs whilst the peptide substrates remain bound to the NRPS machinery, with the interaction between the Oxy enzymes and the NRPS-bound peptide mediated by a unique recruitment domain, known as the X-domain.^23^ The X-domain, found in the final NRPS module of all GPA producing assemblies, is an example of a modified C-domain and the only other reported example of a C/E type domain immediately prior to a terminal thioesterase domain along with the penicillin producing δ-(l-α-aminoadipyl)-l-cysteinyl-d-valine (ACV) synthase.^23^,^24^ Whilst *in vitro* results have been supportive of the X-domain playing a role in the complete enzymatic crosslinking cascade introduced at the heptapeptide stage (and hence on the final NRPS module),^23^,^25^–^28^ *in vivo* experiments provide a different hypothesis favouring hexapeptide cyclisation for all steps before that of the final AB ring insertion, which is catalysed by OxyC.^29^–^34^ This raises the question as to the selectivity of the C-domain connecting modules 6 and 7 of the NRPS machinery, and hence the process of hexapeptide elongation to form the heptapeptide.^32^ Furthermore, within the peptide synthesis machinery itself a phylogenetic analysis of the C-domains within the NRPS machineries in GPA biosynthesis has shown that these are all configured to accept peptides bearing a C-terminally configured d-amino acid residue, despite several (including the C-domain connecting the 6^th^ and 7^th^ modules) actually being in the l-configuration.^35^ Given these unanswered questions surrounding the late steps within GPA peptide biosynthesis, we determined that this would serve as an excellent system in which to address the impact of peptide structure and stereochemistry on the selectivity of condensation domains within the NRPS-mediated biosynthesis of complex peptides.

## Experimental

### General methods

Acetonitrile (ACN), 2-chlorotrityl chloride resin (0.8 mmol g^–1^, Bachem dichloromethane (DCM) (Chem-supply), hydrazine monohydrate 64–65% (Sigma-Aldrich), methanol (Scharlau), *N*,*N*′-diisopropylethylamine (DIEA) (Sigma-Aldrich), Fmoc-amino acids (Merck), l-3,5-dihydroxyphenylglycine (Dpg) (Sigma Aldrich), (1-cyano-2-ethoxy-2-oxoethylidenaminooxy)dimethylamino-morpholino-carbenium hexafluorophosphate (COMU) (Merck), formic acid (FA), triethylamine (TEA) (Sigma-Aldrich), 2,6-lutidine (Sigma-Aldrich), 1,8-diazabicyclo[5.4.0]-undec-7-ene (DBU) (Sigma-Aldrich), *N*,*N*-dimethylformamide (DMF) (Ajax Finechem), trifluoroacetic acid (TFA) (Sigma-Aldrich), triisopropylsilane (TIS) (Sigma-Aldrich), urea (Sigma-Aldrich), NaH_2_PO_4_ (Sigma-Aldrich), NaNO_2_ (Sigma-Aldrich), coenzyme A (Affymetrix).

#### A-domain activity assay

d-fructose-6-phosphate (Sigma-Aldrich), fructose-6-phosphate kinase pyrophosphate-dependent (0.1 U mL^–1^, Sigma-Aldrich), aldolase (1 U mL^–1^, Sigma-Aldrich), triosephosphate isomerase (5 U mL^–1^, Sigma-Aldrich), glycerophosphate dehydrogenase (5 U mL^–1^, Sigma-Aldrich), and NADH (Sigma-Aldrich).

#### Peptide-PCP turnovers and reconstitution assay

Commercial 4-(2-hydroxyethyl)-1-piperazineethanesulfonic acid (HEPES) (Sigma-Aldrich), NaCl (Sigma-Aldrich), MgCl_2_ (Sigma-Aldrich), glucose (Sigma-Aldrich), glucose dehydrogenase (Sorachim), NADH (Sigma-Aldrich).

#### HPLC-MS analysis and purification

For analysis and purification, a HPLC-MS system from Shimadzu (LCMS-2020) was used. UV-spectra were recorded *via* a SPD-20A Prominence Photo Diode Array Detector in analytical mode and *via* a SPD-M20A Prominence Photo Diode Array Detector in preparative mode. Solvents employed were water 0.1% FA and ACN + 0.1% FA for analytical measurements and water + 0.1% TFA and ACN + 0.1% TFA for preparative runs. Turnover analyses were performed using a Waters XBridge®Peptide BEH C18 column, 300 Å, 3.5 μm, 4.6 mm × 250 mm employing a gradient of 5–95% ACN + 0.1% FA in 30 min. Crude peptides were purified using a preparative HPLC Waters XBridge® Peptide BEH C18 OBD™ prep column, 300 Å, 5 μm, 19 mm × 150 mm employing a gradient of 10–40% or 15–45% ACN + 0.1% FA in 30 min.

#### NMR analysis

^1^H NMR analysis spectra were recorded on a Bruker Avance III 600 MHz. Solvent CD_3_CN/D_2_O (20 : 80, v/v).

#### HRMS analysis

HRMS was performed on an Agilent 6220 Accurate Mass LC-TOF system with an Agilent 1200 Series HPLC.

#### UV-vis spectrophotometer

For the A-domain activity assay UV-spectra were recorded using a JASCO V-750 spectrophotometer. For data analysis, the software Prism7 was used.

### Peptide synthesis

For the C-domain selectivity assay, peptides linked to coenzyme A were synthesised according to a previously established protocol.^36^ Fmoc-based SPPS was performed manually on 2-chlorotrityl chloride resin (scale 0.05 mmol, 200 mg). Resin swelling was performed in DCM (8 mL, 30 min), followed by washing with DMF (3×), treatment with 5% hydrazide solution in DMF (6 mL, 2 × 30 min), washing with DMF and capping with a solution of DMF/TEA/MeOH (7 : 2 : 1) (4 mL, 15 min). Amino acid coupling used Fmoc-amino acid (0.06 mmol), COMU (0.06 mmol) and 2,6-lutidine (0.06 mmol, 0.12 M); initial coupling was always performed overnight and a second coupling step was always accomplished to cap unreacted hydrazide groups using BOC-glycine-OH (1 h). Subsequent amino acid couplings were incubated for 40 min. For Fmoc-deprotection, a 1% DBU solution in DMF was used (3 mL, 3 × 30 s). In the last coupling step, a Boc-protected amino acid was always used. The hydrazide peptide intermediate was cleaved from the resin, including ^*t*^Bu and Boc removal, using a TFA cleavage mixture (TFA/TIS/H_2_O, 95 : 2.5 : 2.5 v/v′/v′′, 5 mL) for 1.5 h with shaking at room temperature. The solution was concentrated under nitrogen stream to ∼1 mL and precipitated with ice cold diethyl ether (∼8 mL), followed by centrifugation in a flame-resistant centrifuge (Spintron). All crude hydrazide peptides were purified using a preparative HPLC, and purified hydrazide peptides subsequently converted to CoA-linked peptides. To achieve this, the peptide hydrazide (5 mM) was dissolved in buffer A containing urea (6 M) and NaH_2_PO_4_ (0.2 M), pH 3 (obtained *via* addition of HCl) and the reaction mixture was cooled to –15 °C using a salt/ice bath. In the next step, 0.5 M NaNO_2_ (0.95 eq.) was added to the solution and stirred for 10 min before addition of coenzyme A (1.2 eq., dissolved in buffer A). The solution was adjusted to pH 6.5 by adding KH_2_PO_4_/K_2_HPO_4_ buffer (6 : 94 v/v 1 M, pH 8.0) and stirred for further 30 min on ice with monitoring by LCMS. Final CoA-peptides were purified using preparative RP-HPLC (gradient 10–40% ACN or 15–45% ACN in 30 min) (Table 1).

**Table 1:** Additional peptides synthesised in this study (all synthesised as CoA thioesters, yield indicating conversion of pure hydrazide to CoA conjugate, purity of all peptides > 90%)

Compound	Sequence	Yield [mg]	Yield [%]
**2**	d-Hpg/d-Tyr/l-Hpg/d-Hpg/d-Hpg/l-Phe	0.78 mg	12%
**3**	d-Hpg/d-Tyr/l-Hpg/d-Hpg/d-Hpg/l-4-cyano-Phe	0.54 mg	10%
**5**	d-Hpg/d-Cl-Tyr/l-Hpg/d-Hpg/d-Hpg/l-Cl-Tyr	0.20 mg	7%
**6**	d-Hpg/d-Cl-Tyr/l-Phe/d-Hpg/d-Hpg/l-Cl-Tyr	1.2 mg	8%
**D-1**	d-Hpg/d-Tyr/l-Hpg/d-Hpg/d-Hpg/d-Tyr	0.73 mg	9%
**D-4**	d-Hpg/d-Tyr/l-Phe/d-Hpg/d-Hpg/d-Tyr	2.9 mg	43%
**7**	d-Hpg/d-Tyr/l-Hpg/d-Hpg/d-Hpg	1.0 mg	9%
**8**	d-Hpg/d-Tyr/l-Phe/d-Hpg/d-Hpg	3.7 mg	34%
**9**	d-Hpg/d-Cl-Tyr/l-Phe/d-Hpg/d-Hpg	3.3 mg	60%

### CoA linked hexapeptide teicoplanin (1-CoA) (1.3 mg, 15%)

LC analysis: rt 14.8 min, purity > 90%. ^1^H NMR (600 MHz, CD_3_CN) *δ* 8.57 (s, 1H), 8.57 (s, 1H), 8.33 (s, 1H), 8.27 (s, 1H), 7.09 (d, *J* = 8.7 Hz, 2H), 7.04 (d, *J* = 8.7 Hz, 2H), 6.90 (d, *J* = 8.6 Hz, 2H), 6.80 (d, *J* = 8.5 Hz, 4H), 6.73 (d, *J* = 8.5 Hz, 3H), 6.71 (d, *J* = 8.7 Hz, 2H), 6.66 (d, *J* = 8.7 Hz, 3H), 6.65 (d, *J* = 8.9 Hz, 2H), 6.55 (d, *J* = 8.4 Hz, 2H), 6.51 (d, *J* = 8.5 Hz, 2H), 6.09 (d, *J* = 5.4 Hz, 1H), 6.06 (d, *J* = 5.8 Hz, 1H), 5.28 (s, 1H), 5.25 (s, *J* = 3.7 Hz, 1H), 5.09 (s, 1H), 4.89 (s, 1H), 4.79–4.71 (m, 3H), 4.72–4.68 (m, 1H), 4.16–4.10 (m, 2H), 4.07–4.03 (m, 1H), 3.93 (s, 1H), 3.77 (dd, *J* = 9.7, 5.0 Hz, 2H), 3.46 (dd, *J* = 9.8, 4.5 Hz, 2H), 3.35–3.29 (m, 3H), 3.20–3.08 (m, 3H), 2.96 (dd, *J* = 14.2, 4.5 Hz, 2H), 2.89–2.82 (m, 2H), 2.82–2.74 (m, 3H), 2.72 (dd, *J* = 13.8, 8.5 Hz, 2H), 2.58 (dd, *J* = 13.9, 10.4 Hz, 2H), 2.33–2.27 (m, 3H), 0.85 (s, 3H), 0.69 (s, 3H); HRMS analysis [M – H]^2–^ expected molecular mass 843.7090 (chemical formula C_71_H_18_N_13_O_28_P_3_S^2–^), found 843.7010, *Δ* = 9.4 ppm.

### CoA linked hexapeptide actinoidin (4-CoA) (5.0 mg, 52%)

LC analysis: rt 19.2 min, purity > 90%. ^1^H NMR (600 MHz, CD_3_CN) *δ* 8.58–8.52 (m, 1H), 8.27–8.22 (m, 1H), 7.14–7.06 (m, 5H), 6.99–6.94 (m, 2H), 6.92–6.88 (m, 2H), 6.87–6.83 (m, 2H), 6.83–6.80 (m, 1H), 6.80–6.77 (m, 2H), 6.77–6.74 (m, 1H), 6.74–6.69 (m, 3H), 6.70–6.65 (m, 4H), 6.64 (dd, *J* = 6.1, 2.5 Hz, 2H), 6.52–6.47 (m, 2H), 6.09–6.03 (m, 1H), 5.24–5.19 (m, 2H), 4.90–4.87 (m, 1H), 4.77–4.72 (m, 2H), 4.15–4.12 (m, 2H), 3.95–3.92 (m, 1H), 3.79–3.73 (m, 2H), 3.50–3.44 (m, 2H), 3.35–3.28 (m, 2H), 3.20–3.09 (m, 3H), 2.96 (d, *J* = 14.1 Hz, 2H), 2.88–2.81 (m, 2H), 2.82–2.74 (m, 2H), 2.73–2.61 (m, 5H), 2.59–2.52 (m, 2H), 2.33–2.25 (m, 3H), 2.13–2.09 (m, 1H), 0.83 (s, 3H), 0.69 (s, 3H); HRMS analysis [M – H]^2–^ expected molecular mass 842.7194 (chemical formula C_72_H_82_N_13_O_27_P_3_S^2–^), found 842.7170, *Δ* = 2.8 ppm.

### Protein expression of Tcp12

All Tcp12 constructs (pET-MBP-1c) were co-expressed with the teicoplanin MbtH-like protein Tcp17. This was performed by transforming 50 μL of competent cells with a plasmid encoding Tcp17. Cells were thawed on ice and 1 μL of DNA (20–30 ng for both constructs) was added to the cells. The mixture was incubated for 30 min on ice, before performing a 42 °C heat shock for 10 s and returning the mixture to ice for 5 min. Cells were recovered by adding 750 μL of room temperature SOC media and incubation at 37 °C, 750 rpm for 60 min. After incubation, 450 μL of the mixture were spread onto an antibiotic-selective LB-agar plate having selectivity markers for both plasmids (kanamycin and streptomycin) and incubated overnight at 37 °C. Expression of the Tcp12 constructs was performed in auto-induction media (10 g tryptone, 5 g Na_2_HPO_4_, 3.4 g KH_2_PO_4_, 1.3 g Na_2_SO_4_, 0.24 g MgSO_4_, 5 g glycerol, 0.5 g glucose, 2 g lactose, pH 7.4 adjusted with NaOH per 1 L media) with the media supplemented with the respective antibiotic (kanamycin 50 μg mL^–1^ and streptomycin 50 μg mL^–1^). Inoculation used 1/100 of culture volume of pre-culture. Bacterial growth was performed at 37 °C and 170 rpm for 5 h followed by subsequent reduction in temperature to 18 °C. The culture was then incubated for a further 16–40 h at 18 °C.

### Protein expression of PCP_6_

Transformation of the PCP_6_ domain derived from Tcp11 was performed in BL21(DE) cells following the same procedure as the Tcp12 constructs but without co-expression of an MbtH-like protein. Expression of the PCP_6_ construct (pET-Trx-1b)^37^ was performed in LB-media supplemented with the respective antibiotic (kanamycin 50 μg mL^–1^). Inoculation used 1/100 of culture volume of pre-culture. Bacterial growth was performed at 37 °C and 170 rpm until an OD_600nm_ of 0.6 was reached, upon which the temperature was reduced to 18 °C and protein expression induced by the addition of IPTG (0.1 mM final concentration) followed by incubation for 6 h at 18 °C.

### Protein expression of cytochrome P450 s

OxyB and OxyA (expression vectors pET28 or pET151d) were transformed into *E. coli* KRX and expression took place in LB media supplemented with the respective antibiotic and inoculated by adding 1/100 of culture volume of pre-culture. Bacterial growth took place at 37 °C and 120 rpm until an OD_600nm_ of 0.40–0.45 was reached. Subsequently, the temperature was reduced to 18 °C, δ-aminolevulinic acid (100 μg L^–1^) was added and protein expression was induced through addition of 0.1% (w/v) rhamnose and 0.1 mM IPTG (final concentration); incubation continued overnight at 18 °C (90 rpm).

### Protein purification of NRPS proteins

Cells (PCP_6_ and Tcp12) were harvested using centrifugation (7550 rcf, 10 min, 4 °C). Subsequently, the pellet was resuspended in lysis buffer (50 mM Tris HCl pH 7.3, 50 mM NaCl, 10 mM imidazole, protease inhibitor (Sigma), 15 mL per 2 L culture) and the cells lysed using sonication (Consonic). After centrifugation (38 420 rcf, 40 min, 4 °C) the protein was first purified *via* NiNTA in batch mode (NiNTA wash buffer, 50 mM Tris HCl pH 7.4, 300 mM NaCl, 10 mM imidazole and NiNTA elution buffer, 50 mM Tris HCl pH 7.4, 300 mM NaCl, 300 mM imidazole) and in a final step using size exclusion chromatography (SEC) (Äkta, GE Healthcare, 320 mL Superose 12 column, Buffer 50 mM Tris HCl pH 7.4, 100 mM NaCl). All proteins were flash frozen and stored at –80 °C.

### Protein purification of cytochrome P450 OxyB_bal_

Cell harvesting, lysis and NiNTA purification followed the same protocol as for Tcp12 and PCP_6_. After NiNTA chromatography, the fractions containing protein were pooled and dialysed overnight into anion exchange buffer A (AEX) (20 mM Tris HCl pH 8.0, 50 mM NaCl). Subsequently, AEX chromatography was performed (Äkta, GE Healthcare, 6 mL ResourceQ column). Protein was loaded using AEX buffer A and eluted by applying a gradient from 0–50% AEX buffer B over 20 column volumes (20 mM Tris HCl pH 8.0, 1 M NaCl). As a final purification step SEC was performed using the same buffer as for Tcp12 and PCP_6_. All proteins were flash frozen and stored at –80 °C.

### *In vitro* experiments

#### Online A-domain activity assay

In order to monitor the rate of amino acid activation by ATP of the different Tcp12 constructs, an online activity assay detecting PPi release was used which allows the detection by spectroscopic methods.^38^ The assay can be used with or without an acceptor domain such as the PCP. If it is performed with a PCP-domain present, the PCP can also be converted into the *holo*-form first to allow the loading of amino acids and two rounds of amino acid activation. For the optional PCP-loading reaction 1 μM R4-4 mutant Sfp,^39^ 300 μM PCP and 600 μM CoA in 25 mM Tris, pH 7.4 and 5 mM MgCl_2_ were used. After optional PCP-loading, the A-domain activity assay was performed by using 1 μM of the pre-loaded Tcp12, 0.5 mM ATP and Dpg (0–0.06 mM) in 100 mM Tris, pH 7.4, 1 mM MgCl_2_, 0.1 mM EDTA, 0.2 mM NADH and the components needed for detection (F-6-P = d-Fructose-6-phosphate (3 mM), PPi-PFK = PPi-dependent phosphofructokinase (0.1 U mL^–1^), aldolase (1 U mL^–1^), TPI = triosephosphate isomerase (5 U mL^–1^), GDH = glycerophosphate dehydrogenase (5 U mL^–1^)). The final reaction volume was 0.5 mL.

#### PCP-loading

For the PCP-loading (peptidyl carrier protein) with either CoA or CoA-peptide, a solution containing 60 μM PCP, 50 mM Hepes (pH 7.0), 10 mM MgCl_2_, 50 mM NaCl, 120 μM CoA or CoA-peptide and 6 μM Sfp (PCP : Peptide : Sfp, 1 : 2 : 0.1) was incubated for 1 h at 30 °C. Subsequently, the mixture was washed four times by using Centricon centrifugal concentrators (10k MW cut-off for PCP6 and 100k MW cut-off Tcp12) and buffer (50 mM Hepes, pH 7, 50 mM NaCl).

#### C-domain selectivity assay/P450 crosslinking

After CoA loading steps, peptidyl-PCP_6_ (50 μM), *holo*-Tcp12_ΔTE_2_ (50 μM), ATP (1 mM), MgCl_2_ (10 mM) and amino acid (1 mM) were combined in buffer (50 mM Hepes (pH 7), 50 mM NaCl) and incubated for 3 h at 30 °C, 300 rpm. If the reconstitution assay was combined with P450 turnover, OxyB_bal_ (0.5 μM), PuR (0.66 μM), PuxB A105V mutant (2.5 μM),^40^ glucose (0.33%), glucose dehydrogenase (0.033 mg mL^–1^) and NADH (2 mM) were added. Peptide cleavage from the peptidyl carrier domain was performed through addition of 40% methylamine solution in water (0.5 M) at room temperature for 15 min. Subsequently, the samples were neutralised to pH ∼7.0 with 0.1% formic acid in water and purified *via* solid phase extraction (SPE columns Strata-X-polymeric cartridges, reversed phase). Before the sample was loaded the columns were first conditioned with 1 mL MeOH and activated with 1 mL water. The column material was washed with 1 mL 5% MeOH and elution took place using 0.5 mL of 1% FA in MeOH. The solvent was concentrated *in vacuo* using an Eppendorf concentrator. For HPLC-MS analysis the samples were dissolved in ACN/H_2_O (50 : 50).

### Preparation for *in vivo* experiments

#### Strains and plasmids

*E. coli* XL1-blue was used as general cloning host. *Amycolatopsis balhimycina* DSM5908 is the balhimycin producing wildtype and was used to generate the NRPS mutant *A. balhimycina*_ΔbpsC_X (this study). The inactivation plasmid pESbpsCX (this study) is a derivative of the non-replicative vector pSP1.^41^

#### Media and culture conditions

*E. coli* strains were grown in Luria broth (LB) medium at 37 °C, supplemented with 100 μg mL^–1^ ampicillin when necessary to maintain plasmids. *A. balhimycina* strains were grown in R5 medium^42^ at 30 °C. Liquid/solid media were supplemented with 50 μg mL^–1^ erythromycin to select for strains carrying integrated antibiotic resistance genes.

#### Preparation and manipulation of DNA

Methods for isolation and manipulation of DNA were performed as reported.^42^,^43^ PCR fragments were isolated from agarose gels with QIAquick gel extraction kit (Qiagen, Hilden, Germany). Restriction endonucleases (NEB, Ipswich, MA, USA and Fermentas, St. Leon-Rot, Germany) were used according to their specifications. PCR protocols for amplification of the fragments bpsCXleft, bpsCXright PCRs were performed on a Robo Cycler Gradient 40 thermocycler from Stratagene (La Jolla, CA, USA) with the Expand High Fidelity PCR System (Roche, Grenzach-Wyhlen, Germany). For the amplification of the fragments bpsCXleft and bpsCXright the following PCR conditions were used: initial denaturation (95 °C for 5 min), 30 cycles of denaturation (95 °C for 1 min), annealing (65 °C for 2 min), and polymerisation (72 °C for 2 min), an additional polymerisation step (72 °C for 10 min) at the end. The primers used were as follow: for bpsCXleft (2079 bp): bpsCXleftP1, bpsCXleftP2 and for bpsCXright (1916 bp): bpsCXrightP1, bpsCXrightP2 (Table 2).

**Table 2:** Primer sequences used for preparation of the deletion mutant *A. balhimycina*_bpsCX

Primer	Sequence
*bpsCXleftP1*	TTTATAGCATGCCGGAACTCCTCGCACTACCCGTTCAC
*bpsCXleftP2*	AATAATTCTAGAATCGGCCAGCAGCCAGGCACG
*bpsCXrightP1*	TTTATATCTAGATTCACCCGGGCGCTCGCCCTG
*bpsCXrightP1*	AATAATGAGCTCCTCCTCGAACACTGCACAAGGTCC

#### Construction of the inactivation plasmid pESbpsCX

**Fig. 3:**
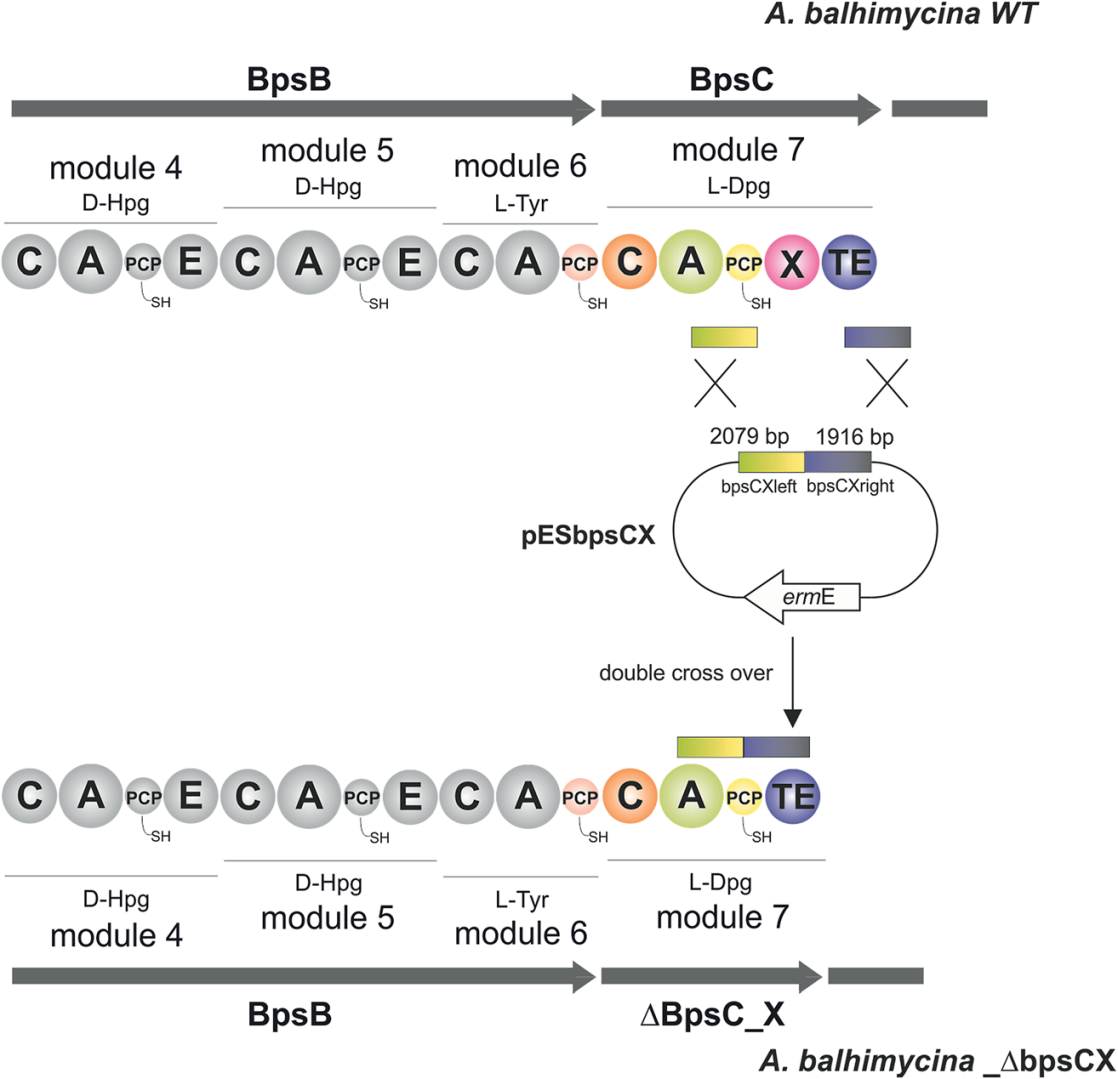
Construction of the mutant *A. balhimycina*_ΔbpsCX. Schematic representation of the deletion of the X domain. Domain arrangement shown for the balhimycin NRPS. NRPS domain descriptions: C, condensation; A, adenylation; PCP, peptidyl carrier protein; E, epimerisation; X, P450 (Oxy) recruitment; TE, thioesterase. *ermE*, erythromycin resistance gene. bpsCXleft (2079 bp): red; bpsCXright (1916): green. To obtain the deletion mutant *A. balhimycina*_ΔbpsCX a double crossover *via* homologous recombination is required.

pESbpsCX was constructed for the inactivation of the X domain of module 7 (*bpsC*). To this end, the fragments bpsCXleft, bpsCXright were amplified by PCR. The bpsCXleft, bpsCXright fragments were cloned into the pDrive vector (Qiagen) (bpsCXleft/pDrive; bpsCXright/pDrive). Subsequently, both fragments were cloned into the non-replicative vector pSP1 using SphI and XbaI for bpsCXleft and XbaI and SacI for bpsCXright to obtain pESbpsCX (Fig. 3).

#### Direct transformation of *A. balhimycina*

For transformation of *A. balhimycina*, a modified transformation method was used as described previously.^41^

#### “Stress” protocol

The stress treatment was essentially used as described previously.^44^,^45^ For further fragmentation, protoplast were generated as described by Thompson *et al.*^45^ After storage on ice (10 min), 100 μL of appropriate dilutions (10^–1^ to 10^–4^) were plated on R5 agar plates. After incubation at 30 °C for 10–14 days, the colonies were used for further investigation.

#### Determination of balhimycin biosynthesis

Balhimycin production was determined by bioassays using *Bacillus subtilis* ATCC6633 as a test organism and cell-free supernatants of *A. balhimycina* strains grown in R5 medium.

### HPLC-ESI-MS measurements

Prior to HPLC-MS analysis the extracts were concentrated and desalted by solid phase extraction. To this end, a 1 g chromabond C_18_ cartridge (Macherey & Nagel, Düren, Germany) was conditioned with methanol (MeOH, 1 column volume) and H_2_O (1 column volume), after which 2 mL of the respective extracts were applied to the column. The column was washed with H_2_O (3 column volumes) and eluted with MeOH (2 column volumes). The concentrated extracts were then dried in a Speedvac (Genevac EZ-2 MK2, Ioswich, United Kingdom), resuspended in 200 μL 50% MeOH and subjected to HPLC-ESI-MS as described below. The HPLC-MS measurements were conducted on an Exactive ESI-Orbitrap-MS (Thermo Fisher Scientific, Bremen, Germany) connected to an analytical Agilent 1200 HPLC system (Agilent, Waldbronn, Germany) equipped with a GRACE Grom-Sil120 ODS-4 HE column (50.0 × 2.0 mm; Grace, Deerfield, IL, USA). The mobile phase consisted of H_2_O as solvent A and acetonitrile as solvent B, both acidified with 0.1% formic acid. The gradient increased linearly from 5–100% solvent B over 17 min. Measurements were conducted in positive ionization mode. Data analysis was performed using the Thermo Xcalibur 2.2 software.

## Results and discussion

### Reconstitution of final GPA NRPS module encoded by the Tcp12 protein

**Fig. 4:**
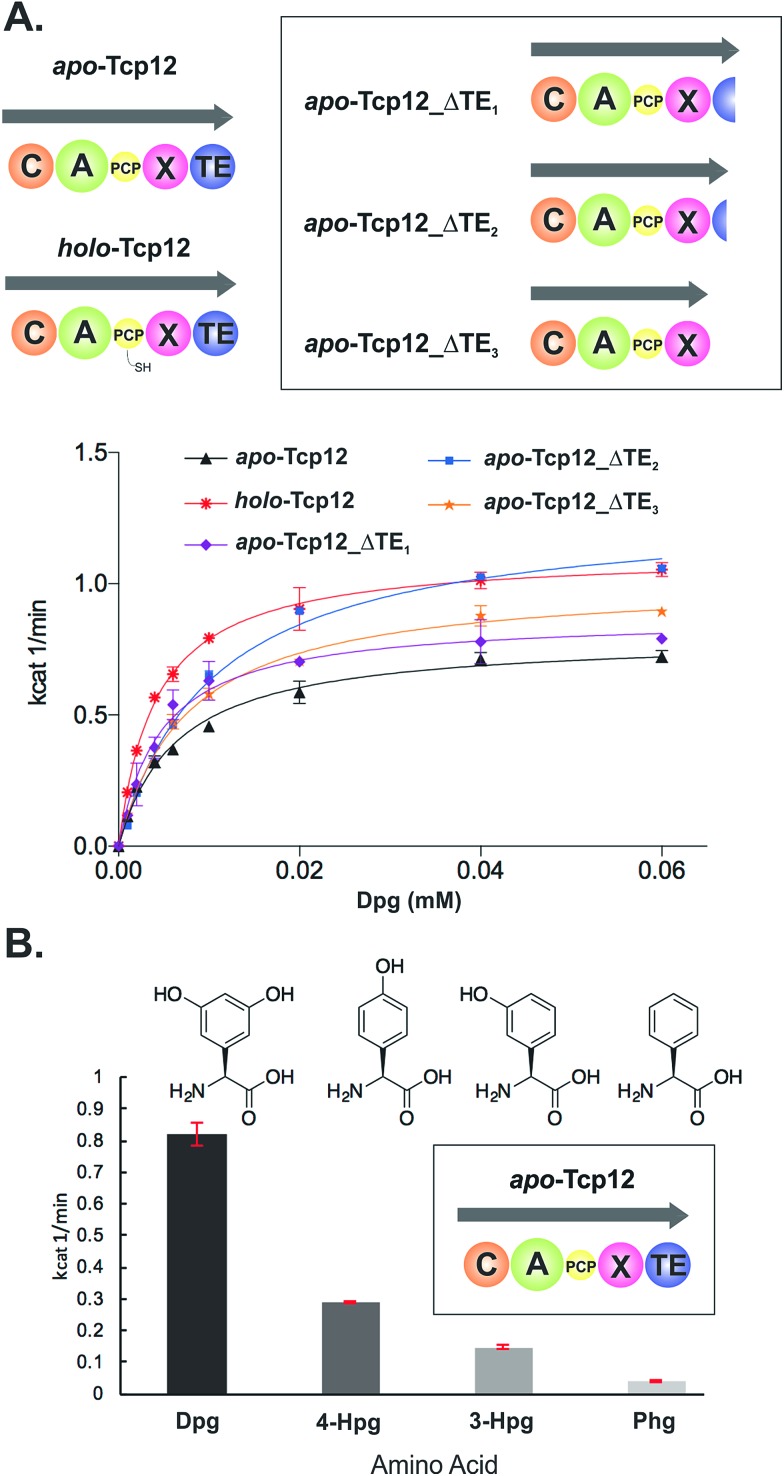
Characterisation of the amino acid selection and activation characteristics of the final NRPS module from teicoplanin biosynthesis (Tcp12). (A) Alternate constructs of Tcp12, the final module from the teicoplanin NRPS, were designed in order to remove the C-terminal TE-domain in order to prevent unwanted peptide hydrolysis during subsequent C-domain assays (Tcp12_ΔTE_1_, Tcp12_ΔTE_2_, Tcp12_ΔTE_3_). NRPS domain descriptions: C, condensation; A, adenylation; PCP, peptidyl carrier protein; X, P450 (Oxy) recruitment; TE, thioesterase. (B) Amino acid selectivity of the A-domain of the *apo*-Tcp12 protein for the natural substrate Dpg (l-3,5-dihydroxyphenylglycine) as well as related Phg substrates (4-Hpg: l-4-hydroxylphenylglycine; 3-Hpg: l-3-hydroxylphenylglycine; 4-Hpg: l-phenylglycine).

In order to study the final condensation domain within GPA biosynthesis it was first essential to reconstitute the activity of the final module within the NRPS machinery – specifically encoded by the protein Tcp12 in teicoplanin biosynthesis (Fig. 2).^46^ This module consists of 5 domains and exhibits the C-A-PCP-X-TE architecture conserved for GPA producing NRPS systems bearing the specific P450 recruitment (X)-domain.^23^,^35^ In order to study this module, we initially identified that overexpression in *E. coli* was enabled by the co-expression of the MbtH protein Tcp17 from the teicoplanin gene cluster, together with the expression of Tcp12 as an MBP fusion protein to improve protein yield.^37^ Expression without an MbtH protein led to significant degradation of the protein during expression, whilst co-expression of the other MbtH protein in the teicoplanin gene cluster (Tcp13) did not provide the same overall yield as Tcp17. Following a two-step purification protocol employing sequential Ni-affinity and gel filtration steps, the catalytic competence of the module was tested both in terms of the ability to convert the PCP from the *apo* to the phosphopantetheine bearing *holo* form and the subsequent ability of the neighbouring A-domain to select, activate and load amino acid substrates onto this PCP domain. First, reconstitution of the *holo*-PCP state was successfully accomplished using the promiscuous phosphopantetheinyl transferase Sfp (R4-4 mutant).^39^ Subsequently, A-domain activity was tested for the natural substrate (*S*)-3,5-dihydroxyphenylglycine (Dpg) using a coupled enzymatic activity assay, which allows an assessment of the rate of activity of the A-domain as well as the number of A-domain cycles performed (based on the amount of PPi released, Fig. 4).^38^ This assay showed that the A-domain within the final module encoded by Tcp12 was active and able to load Dpg onto the neighbouring PCP domain within the module at a rate of 0.8–1.1 min^–1^ (Table 3 and Fig. 4). This rate is comparable to that seen for the only other A-domain from teicoplanin to have been characterised (1.6 min^–1^ for Dpg activation by NRPS module 3, encoded by the protein Tcp10),^38^ and is comparable to the rates reported for other complex assembly lines (pyochelin NRPS: ∼2 min^–1^;^45^*Pseudomonas* virulence factor NRPS: 3.4 min^–1^;^47^ yersiniabactin NRPS/PKS hybrid: ∼1.4 min^–1^;^46^ 6-deoxyerythronolide B PKS: 1 min^–1^).^47^ The observed rate of Tcp12 A-domain activity is, however, significantly slower than the observed rate peptide cyclisation enzymes that should act subsequent to heptapeptide bond formation (each ∼10 min^–1^).^26^ The slower rate of amino acid activation – and hence peptide bond formation – would allow the production rate for linear GPA peptides to be well matched to their complete maturation (3–4 cyclisation steps) prior to the selective cleavage of the completely cyclised peptide from the NRPS through the actions of the TE domain.^48^

**Table 3:** Michaelis–Menten kinetics determined for the different Tcp12 constructs

Tcp12 construct	Tcp12 concentration [μM]	*k*_cat_ [min^–1^]	*K*_m_ [mM]
Tcp12 *holo*-form	1	1.12 ± 0.01	0.004 ± 0.0001
Tcp12 *apo*-form	0.5	0.87 ± 0.03	0.005 ± 0.0005
Tcp12_ΔTE_1_	0.5	0.79 ± 0.02	0.006 ± 0.0006
Tcp12_ΔTE_2_	0.5	1.28 ± 0.04	0.01 ± 0.0009
Tcp12_ΔTE_3_	0.5	1.02 ± 0.02	0.008 ± 0.0006

Before utilising the Tcp12 protein for peptide bond formation assays we were concerned about the potential interference of the C-terminal thioesterase (TE) domain in C-domain assays. Whilst this domain has been shown to have a preference for activity against completely crosslinked PCP-bound peptides, hydrolysis of linear peptide has also been demonstrated for this domain.^48^ Given that such hydrolysis would not allow us to assess the possible role of peptide hydrolysis performed by the C-domain, we designed, expressed and purified three C-terminally truncated forms of Tcp12 (Fig. 4A). These constructs either removed the minimal TE-domain (Tcp12_ΔTE_1_), the extended TE-domain (Tcp12_ΔTE_2_) or the complete linker-TE region beyond the X-domain (Tcp12_ΔTE_3_). All proteins could be expressed and purified as for the wildtype protein, and gratifyingly the activity of the A-domain within all constructs in their *apo*-PCP form was comparable to that of the *apo*-PCP wildtype protein (Table 3). For ongoing C-domain experiments, we then selected the construct Tcp12_ΔTE_2_ as this was the construct with the highest rate of amino acid activation. We also tested the acceptance of other phenylglycine substrates (Fig. 4B) in comparison to the natural, preferred Dpg substrate by *apo*-Tcp12. This showed that singly hydroxylated 4- and 3-Hpg substrates were also accepted by this A-domain, albeit at ∼40% and ∼20% of the Dpg rate respectively, whilst Phg was a poor substrate for this A-domain.^49^ This result is somewhat surprising given the presence of 4-Hpg residues within GPAs (and hence the presence of this amino acid within the producer strain), although the activation of 4-Hpg does explain the presence of modified (*i.e.* Hpg-containing) GPAs in producer strains in which Dpg production had been abolished.^32^ This result was also useful in the context of our current study, as it would allow us to probe the effect of A-domain rate upon the production of peptides by the neighbouring C-domain once this had been reconstituted (see below).

### C-domain displays broad substrate selectivity and stereochemical tolerance

**Fig. 5:**
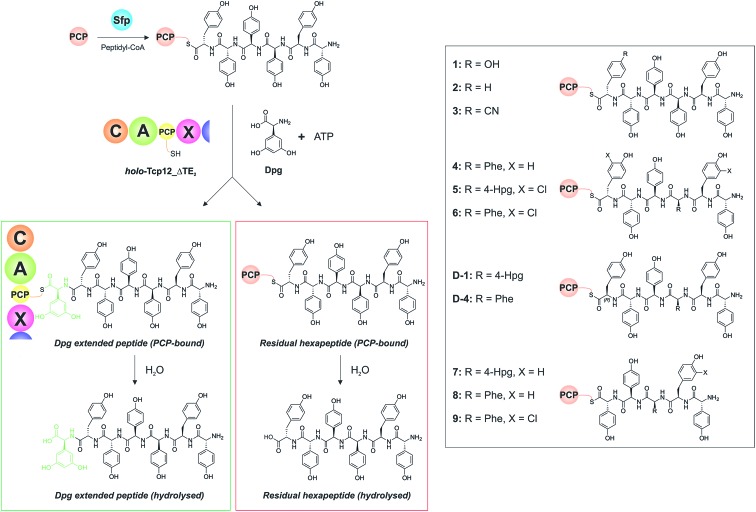
Condensation domain assay for the final module of the teicoplanin NRPS. Initially, peptidyl-CoA substrates prepared by solid phase peptide synthesis are loaded onto the isolated PCP domain from the preceding module using the promiscuous phosphopantetheinyl transferase Sfp (top left), after which these substrates are then added to the Tcp12_ΔTE_2_ construct along with Dpg and ATP in order to assess the formation of Dpg-extended peptide products. Products peptides are extended by the addition of Dpg through the actions of Tcp12_ΔTE_2_ (green box, residual starting peptide shown in the red box). All peptides can either remain PCP-bound at the end of the assay (where they are then analysed as their methylamides through the addition of methylamine) or they can be hydrolysed from the PCP. Peptide structures synthesised and used as substrates in these assays are shown in the boxed area on the right of the figure (**1–9**, **D-1**, **D-4**). NRPS domain descriptions: C, condensation; A, adenylation; PCP, peptidyl carrier protein; X, P450 (Oxy) recruitment.

With a functional, truncated Tcp12_ΔTE_2_ protein in hand, we then turned to the characterisation of the C-domain within this construct. To this end, we synthesised 11 different peptides (Table 4, SI1,† and Fig. 5), initially based on a range of potential hexapeptide substrates as their coenzyme A (CoA) thioesters using our reported Fmoc-based solid phase synthesis route.^36^,^50^ The peptides conformed to the sequence of teicoplanin (**1**) and were designed to explore the tolerance of the C-domain for modifications in the peptide structure at various positions throughout the peptide. These included peptides in which the C-terminal Tyr residue was exchanged for other amino acid residues (Phe (**2**), 4-CN-Phe (**3**)), the variable amino acid at position 3 was exchanged for the type-II GPA sequence (Phe (**4**), actinoidin), and/or the Tyr residues in the peptide were exchanged for chlorinated Tyr residues (**5**, **6**) (Fig. 5). Furthermore, we synthesised hexapeptides in which the C-terminal Tyr residue was present in the non-natural d-configuration to explore the stereochemical selectivity of the C-domain (**D-1**, **D-4**), and also truncated pentapeptides (**7–9**) to test the effect in alterations in peptide length on peptide bond formation. At this point, we cloned, expressed and purified the PCP domain from the preceding NRPS module (module 6) as a thioredoxin (Trx)-fusion protein^37^ to be able to use this protein to present these peptides to the C-domain. Use of the PCP-domain proved essential for this assay, as there was no activity of the C-domain detected when isolated CoA peptides were used. Peptidyl-PCP substrates were then prepared for C-domain activity assays by loading the peptidyl-CoAs onto the *apo*-PCP domain using the promiscuous R4-4 Sfp mutant.^39^ The C-domain activity assays were performed in triplicate, and utilised a 1 : 1 mixture of loaded peptidyl-PCP and *holo*-Tcp12_ΔTE_2_, along with Dpg and ATP to generate the required C-domain aminoacyl-PCP acceptor substrate (Fig. 5).

**Table 4:** Summary of all results from Tcp12 reconstitution with different peptides (**1–9**, **D-1**, **D-4**) and the adenylation domain substrates Dpg and ATP. All peptides were presented bound to PCP_6_

Substrate	Yield^*a*^ [%]			
	Dpg extended peptide, PCP-bound^*b*^	Dpg extended peptide, hydrolysed^*c*^	PCP_6_-bound peptide^*d*^	Hydrolysed starting peptide^*e*^
**1**	52.8 ± 1.9	1.6 ± 0.1	40.8 ± 1.3	4.9 ± 0.9
**2**	64.2 ± 3.2	4.2 ± 1.1	16.8 ± 0.8	14.8 ± 1.8
**3**	76.4 ± 0.25	4.6 ± 0.81	7.7 ± 0.37	11.3 ± 0.7
**4**	73.9 ± 5.1	4.8 ± 0.6	11.0 ± 1.7	10.3 ± 5.6
**5**	47.8 ± 2.2	3.9 ± 0.4	45.4 ± 2.4	3.0 ± 0.3
**6**	61.3 ± 1.4	7.0 ± 1.3	19.7 ± 0.5	11.9 ± 0.3
**7**	8.6 ± 0.4	1.4 ± 0.1	80.5 ± 2.2	9.6 ± 2.5
**8**	48.5 ± 11.3	2.6 ± 1.1	41.1 ± 12.1	7.8 ± 0.9
**9**	64.3 ± 1.3	2.3 ± 0.2	27.9 ± 1.0	5.5 ± 0.3
**D-1**	35.2 ± 8.1	8.6 ± 7.5	39.2 ± 10.8	17.1 ± 8.2
**D-4**	55.9 ± 2.7	6.1 ± 0.2	27.3 ± 2.5	10.7 ± 0.2

^*a*^Total yield of extended peptide is based on the percentage reduction of initial hexapeptide peak from initial starting material. The hydrolysed/PCP-bound fractions for each peptide length is determined by dividing the area for each peak by the sum of both peptide peaks.

^*b*^Elongated product cleaved through the use of methylamine to cleave the PCP_7_-bound thioester.

^*c*^Elongated product hydrolysed from PCP_7_ domain of Tcp12 construct during the course of the reaction.

^*d*^PCP_6_-bound peptide substrate cleaved with methylamine.

^*e*^Starting peptide hydrolysed from PCP_6_ during the course of the reaction.

Initial results using the teicoplanin-like hexapeptide (**1**) demonstrated that the assay worked well, with more than 50% conversion into the heptapeptide determined (Table 4). This result also showed that the entire module 6 was not required to support peptide bond formation, thus greatly simplifying the assay. Modifications of the peptide, either the C-terminal (6^th^ from the peptide N-terminus) residue (**2-3**) or variable residue 3^rd^ from the peptide N-terminus (**4**) maintained (and indeed improved) high levels of peptide formation. The chlorination state of the peptide (**5-6**) did not dramatically alter peptide formation in any case except for the pentapeptides, which showed significant variability in peptide yield depending on the sequence used (**7–9**). The tolerance for peptide chlorination is in keeping both with the reported activity of the Oxy enzymes and the timing of GPA chlorination during peptide synthesis,^25^ which has been demonstrated to occur on PCP-bound amino acids.^51^

These results are in keeping with the general role ascribed to C-domains as merely stereochemical gatekeepers, with there being little need for C-domains to be highly selective for the peptide substrates themselves due to the selectivity of amino acid selection performed by A-domains. Unexpectedly, however, peptides bearing the C-terminal Tyr residue in the incorrect d-configuration (**D-1**, **D-4**) remained effective substrates for the C-domain, with only a 20% reduction in yield in these cases. This result is certainly unusual for a domain believed to be responsible for stereochemical selection during peptide bond synthesis, although a hypothesis explaining this result can be made based on the evolutionary history of the GPA NRPS machinery.^35^ Phylogenetic analysis of GPA C-domains has shown that all these C-domains cluster in the ^D^C_L_ C-domain clade, and hence that all these domains initially accepted peptides bearing a d-configured C-terminal residue. As the residues found in positions 3 and 6 of most GPAs are l-configured (Fig. 2), it can be anticipated that the C-domains in modules 4 and 7 must have evolved to accept peptides with an l-configured C-terminal residue. Our results from the module 7 C-domain indicate that this evolution towards acceptance of l-configured substrates has not led to the significant loss of activity for d-configured peptide substrates. This again is attributable to the specificity of A-domains, albeit this time for l-configured residues, for d-configured residues within NRPS peptides typically require an epimerisation (E)-domain with in the module to affect this change in stereochemistry. As there is no E-domain within module 6 of modern GPA NRPS assembly lines, this means that there is no enzymatic means to generate the d-configured peptide substrate, and hence no need for the downstream C-domain to select against this substrate during synthesis. This is an important result, for it suggests that the evolutionary history of C-domains within modern NRPS clusters can have important and unexpected effects on their stereochemical selectivity.

### A-domain rate is coupled to the efficiency of peptide bond formation in neighbouring C-domains

**Fig. 6:**
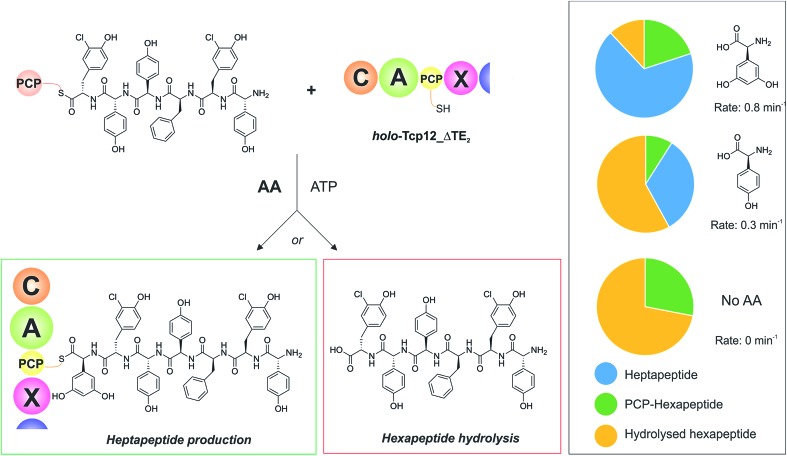
Determining the effect of downstream A-domain rate on upstream C-domain peptide bond formation by varying the amino acid provided during the peptide bond forming step. Summary of the assay (left), in which peptide **4** was loaded onto PCP_6_ and used to reconstitute heptapeptide bond formation in the presence of the natural A-domain amino acid substrate Dpg, a substrate activated ∼2.5× more slowly (4-Hpg) and no amino acid substrate. Results (box on right) show that an increase in hydrolysed hexapeptide starting material (shown in yellow; residual PCP-bound hexapeptide shown in green) correlates to the presence of a poorer (or no) suitable A-domain substrate, with a decrease in extended peptide product (shown in blue).

With an understanding of the specificity of the C-domain for peptidyl-PCP donor substrates, we then turned to investigate the effect of utilising different aminoacyl-PCP acceptor substrates, specifically 4-Hpg (Table 5). We were particularly interested in this residue as our initial A-domain characterisation efforts had showed that this residue was accepted at a reduced rate compared to the natural Dpg substrate (Fig. 4), and we wanted to utilise this reduction in rate to explore the potential coupling between the rate of downstream A-domains with upstream C-domain activity. Given that the A-domain activation cycle has been demonstrated to play a major role in the positioning of the neighbouring PCP domain relative to upstream or downstream domains,^52^,^53^ we hypothesised that a reduction in the rate of this A-domain cycle could cause deleterious effects on hydrolysis of the upstream donor peptide due to it being bound to the C-domain in the absence of aminoacyl-PCP acceptor. We therefore tested this hypothesis and compared the levels of heptapeptide produced as well as hexapeptide hydrolysed in our assay using either Dpg or 4-Hpg as acceptor substrates (Table 5, Fig. 6).

**Table 5:** Summary of A-domain rates for Dpg and 4-Hpg and product yields gained from the C-domain activity assay

Time [min]	Yield^*a*^ [%]			
	*k*_cat_ [min^–1^]	Dpg-extended peptide products^*b*^	PCP_6_-bound peptide^*c*^	Hydrolysed starting peptide^*d*^
Dpg	0.8 ± 0.02	68 ± 0.6	20 ± 0.3	12 ± 0.2
4-Hpg	0.3 ± 0.004	33 ± 2.7	9 ± 1.2	58 ± 3.3
No AA	—	0	28^*e*^ ± 0.1	72^*e*^ ± 1.7
No AA/Tcp12_ΔTE_2_	—	—	92 ± 0.5	8 ± 0.5

^*a*^Total yield of extended peptide is based on the percentage reduction of initial hexapeptide peak from initial starting material.

^*b*^Sum of elongated products either cleaved through the use of methylamine to cleave the PCP-bound thioester hydrolysed from the PCP_7_ domain of the Tcp12 construct during the course of the reaction.

^*c*^PCP_6_-bound peptide substrate cleaved with methylamine.

^*d*^Starting peptide hydrolysed from PCP_6_ during the course of the reaction.

^*e*^Reaction included co-incubation with OxyB_bal_ enzyme, so these values also include a very small proportion of monocyclic peptide starting material (<5%).

Our results showed that there was a significant reduction in heptapeptide produced when using 4-Hpg displaying reduced A-domain activation rate (33%) as compared to assays containing Dpg (68%), which closely matches the reduction in rate for the A-domain (2.5× reduction in rate, 2.1× decrease in peptide formation) (Table 5, Fig. 6). Furthermore, the reduction in heptapeptide production is due to a significant increase in the hydrolysis of the hexapeptide in the 4-Hpg containing assays (58% *vs.* 12%). This supports the hypothesis that interrupting the coupling of C-domain and A-domain activity can cause a significant reduction in effective peptide production by such NRPS systems due to hydrolysis of C-domain bound peptides. We tested exclusion of an amino acid acceptor substrate from our C-domain assays and demonstrated that there was significant hydrolysis of the hexapeptide donor substrates in this case (72%) that was significantly above that of background peptide hydrolysis (8%) in the absence of the C-domain. This result further supports the hypothesis that a decoupling of A-domain activity from the downstream C-domain leads to hydrolysis of the peptide by the C-domain in these cases through hydrolysis (Table 5 and Fig. 6). These results help to explain the results of NRPS A-domain modification experiments *in vivo*, which have shown that such modified assembly lines can produce significant amounts of truncated peptide immediately prior to incorporation of the modified amino acid residue.^17^,^18^ Rather than this being ascribed to the effects of C-domain selectivity for the modified peptide (which our results have shown to be rather flexible), our hypothesis would instead suggest that peptide hydrolysis is a result of the slow formation and hence delivery of the aminoacyl-PCP acceptor substrate in these cases, which is caused by the introduction of a modified A-domain with a slower amino acid activation rate than the original A-domain. This strongly argues for the need to test the properties of such modified constructs *in vitro* prior to engaging in *in vivo* NRPS redesign, which can have unintended deleterious consequences for NRPS efficiency if the rates of activity of modified A-domain domains are significantly slower than those present in the wildtype system. Studies have noted that the substrate selectivity of A-domains observed *in vitro* can be altered by the presence or absence of the adjacent C-domain:^54^,^55^ our results now indicate that C-domain activity is closely coupled to that of the A-domain, which more than ever speaks to the need to characterise complete NRPS modules to truly assess their selectivity and function.

### Relationship between peptide bond formation and the X-domain mediated P450-cyclisation cascade: the timing of peptide cyclisation

**Fig. 7:**
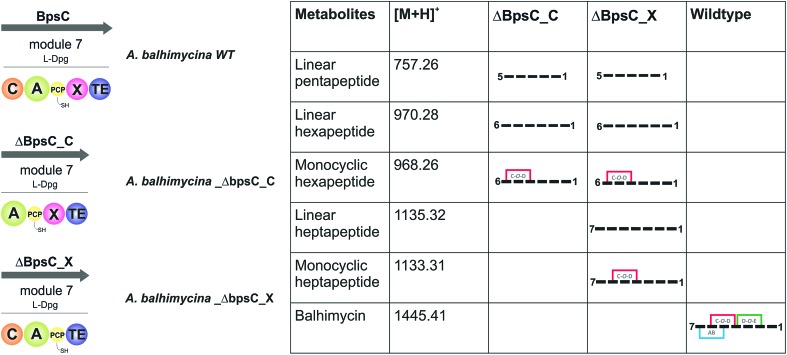
Analysis of the roles of the C- and X-domains within the final NRPS module from balhimycin biosynthesis as assessed through the isolation and analysis of the peptide products formed by the resultant mutant producer strains in which these domains had been removed. Results show that the initial cyclisation step performed by OxyB can occur on the peptide at the hexapeptide stage, which raises the question of cyclisation *vs.* heptapeptide formation during GPA biosynthesis. NRPS domain descriptions: C, condensation; A, adenylation; PCP, peptidyl carrier protein; X, P450 (Oxy) recruitment; TE, thioesterase.

GPA biosynthesis requires the essential, late stage modification of the peptide by cytochrome P450 enzymes to introduce crosslinks between the side chains of specific amino acids within the NRPS-bound peptide (Fig. 2).^21^ Whilst the X-domain present in the final module of all GPA-producing NRPS machineries has been implicated in recruitment of these P450 enzymes, the exact time of the cyclisation events within GPA biosynthesis are somewhat unclear.^23^ Given that all crosslinks prior to the final AB ring, catalysed by OxyC, can theoretically be installed at the hexapeptide stage and that such species had been identified from *in vivo* experiments investigating GPA biosynthesis in *A. balhimycina* and *Streptomyces toyocaensis*,^29^–^34^ we wanted to explore the cyclisation cascade in context of the final peptide bond formation step to clarify the exact timing of the GPA cyclisation cascade. To this end, we turned to the balhimycin producer *A. balhimycina*, the most widely studied GPA assembly line *in vivo* due to it being the sole GPA producer that was able to be manipulated for many years.^32^–^34^,^41^ We first created two modified GPA producer strains in which either the C-domain or the X-domain from the final NRPS module were deleted (Fig. 3) and searched for any evidence that cyclisation could occur at the hexapeptide state (Fig. 7). Analysis of the culture filtrates from the C-domain deletion strain showed the absence of heptapeptides and the presence of both linear and monocyclic hexapeptides, supporting the ability of NRPS-hexapeptides to be modified by the GPA cyclisation cascade (Fig. SI1 and SI2†). Analysis of the X-domain deletion strain showed similar hexapeptide results as the C-domain deletion strain, although the presence of linear and monocyclic heptapeptides was now also detected due to the ability of this NRPS to elongate hexapeptides (Fig. 7). All peptides detected contained a Cl-Tyr_2_ residue and hexapeptide and heptapeptide species also containing a Cl-Tyr_6_ residue, which is in keeping with GPA chlorination occurring during peptide synthesis on specific PCP-bound amino acid residues.^51^

The presence of monocyclic peptides in these *in vivo* studies raised questions about the timing and substrate specificity of the OxyB_bal_ enzyme during peptide synthesis. Whilst *in vitro* activity assays have shown that all OxyB homologues tested to date display the highest level of activity on substrates where the X-domain is present, some OxyB homologues also display reasonable activity against PCP-bound peptide substrates in the absence of the X-domain (including OxyB_bal_ and OxyB_van_).^23^,^26^,^36^,^56^–^61^ As the peptide synthesis machinery is likely to be stalled because of the modified NRPS assembly line in these mutant strains, this would provide an opportunity for relatively slow processes (such as OxyB activity against peptides bound to PCP-domains without a neighbouring X-domain) to occur that are not typically involved in the peptide synthesis process. The lack of production of bicyclic peptides by these mutant strains – which is theoretically possible following OxyB activity – matches recent data from *in vitro* activity assays that show a strict dependence on the presence of the X-domain for the activity of the bicyclisation enzyme OxyA. Given this, we concentrated on understanding the timing of the initial peptide cyclisation step performed by OxyB. In order to explore whether the appearance of monocyclic hexapeptides was an on-pathway process or rather was being caused by the stalling of the NRPS machinery in modified producer strains, we then performed several *in vitro* experiments to characterise the relative acceptance of the C-domain from the final GPA NRPS module for linear and monocyclic peptides.

**Fig. 8:**
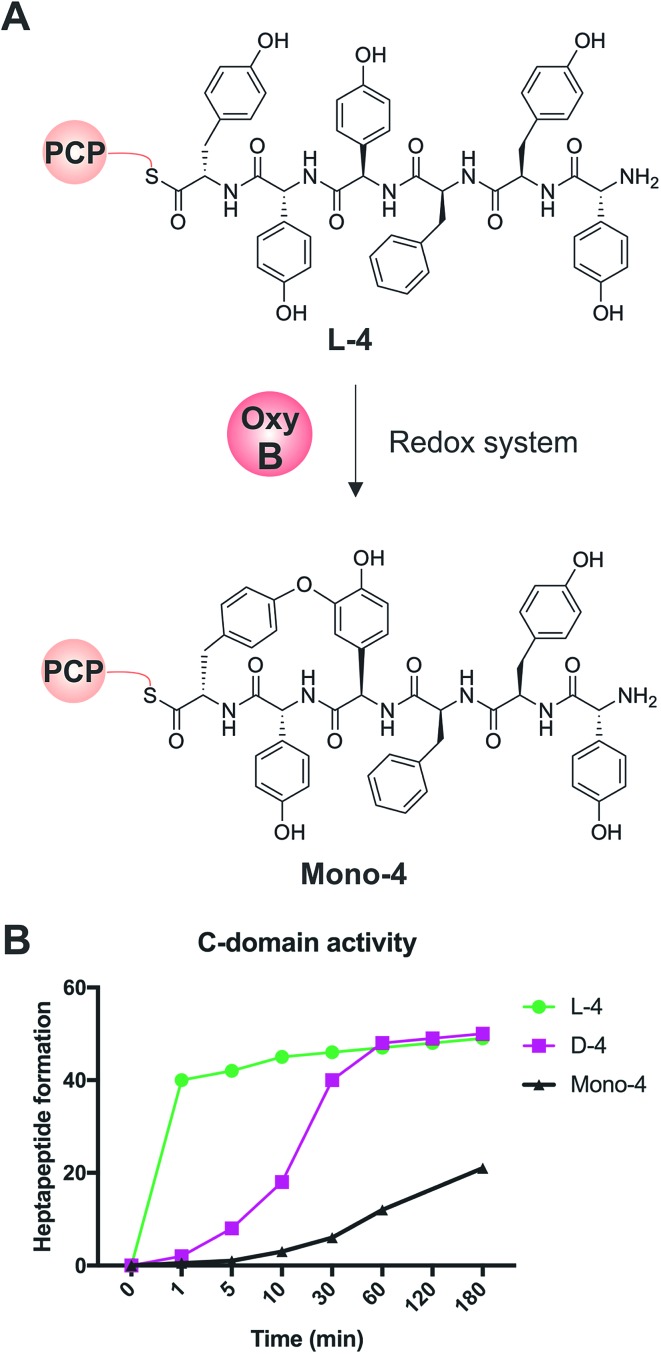
Analysis of the acceptance of the Tcp12 C-domain for different hexapeptide substrates, either the standard linear hexapeptide (**L-4**), a peptide bearing altered stereochemistry at the C-terminal residue (**D-4**) and a monocyclic version of **L-4** (**Mono-4**) formed through the actions of OxyB_van_. (A) Initial formation of **Mono-4** was performed on PCP_6_ using OxyB_van_ and an appropriate redox system. (B) Comparison of the rate of cyclisation of these three peptides under identical reaction conditions shows that the **L-4** peptide is accepted significantly faster than the d-configured form of this peptide (half conversion at 30 seconds *vs.* 15 minutes), whilst the cyclised peptide **Mono-4** is only accepted for peptide extension at a very slow rate (half conversion > 200 minutes).

We first confirmed reported results that OxyB-catalysed peptide cyclisation activity *in vitro* (Fig. 8A) was significantly reduced for PCP substrates alone as compared to PCP-X di-domain substrates (Table 6)^23^,^56^,^57^,^60^ even when using the OxyB homologue from balhimycin activity that has high levels of reported activity using PCP-bound hexapeptides as substrates. We then showed that the hydrolysis of linear hexapeptides in the presence of the truncated Tcp12_ΔTE_2_ construct was much faster than competitive OxyB_bal_ activity against the PCP-bound linear hexapeptide (see Table 5, entry 3). However, to fully test the ability of the C-domain to accept monocyclic peptide substrates, we pre-incubated OxyB_van_ with the PCP-bound hexapeptide **4** to generate significant quantities of the PCP-bound monocyclic hexapeptide (**Mono-4**, ∼75%); it should be noted, however, that this cyclisation activity is significantly slower and delivers lower final yields that when the X-domain is also present, which is in keeping with the importance of the X-domain for Oxy recruitment.^56^–^61^ We then included **Mono-4**-PCP_6_ into our established C-domain activity assay and could show that whilst PCP-loaded **Mono-4** is able to be converted into the monocyclic heptapeptide by the C-domain, this is a very slow process (only ∼20% complete after 3 hours). In comparison, we determined the relative rates of heptapeptide formation for both comparable linear l- and d-configured hexapeptides (**L-4** and **D-4**) and showed that the PCP-bound monocyclic peptide **Mono-4** was ∼3 orders of magnitude slower than the PCP-bound l-configured linear peptide and ∼2 orders of magnitude slower than the PCP-bound d-configured linear peptide (Fig. 8B). Given this dramatic difference in C-domain activity between the linear and monocyclic peptides, our results strongly suggest that all GPA crosslinking in a complete NRPS assembly line occurs on the final NRPS module and is mediated by the X-domain. The presence of monocyclic peptides in modified GPA producer strains can be explained by the ability of OxyB_bal_ to cyclise PCP-bound peptides in the absence of a neighbouring X-domain at a much lower rate than when the X-domain is present: given that the GPA-producing NRPS machinery is effectively stalled in the modified producer strains, the products of these slow reactions now become visible. These results greatly help with the interpretation of results from *in vivo* experiments using modified producer strains, which often display unexpected modified intermediates (in this case, cyclised hexapeptides). Our *in vitro* assays show that the detection of such intermediates can occur as a result of slow processes that in a fully functional NRPS system are unable to effectively compete with the on-pathway peptide synthesis process. Such possibilities must therefore be kept in account therefore when interpreting the results of *in vivo* experiments that affect the NRPS assembly process.

**Table 6:** Comparison of cyclisation efficiency of OxyB_bal_ against hexapeptide **4** bound to either the standalone PCP_6_ domain or loaded onto Tcp12 *via* the PCP_7_ domain. Results show that the final module is much better at supporting peptide cyclisation by OxyB enzymes than PCP_6_ alone

Substrate	Yield [%]	
	PCP-bound **Mono-4**^*a*^	PCP-bound **4**^*b*^
Tcp12 (**4**)	60	40
PCP_6_ (**4**)	33	67

^*a*^PCP-bound **Mono-4** = sum of monocyclic product cleaved with methylamine from the relevant PCP-domain.

^*b*^PCP-bound **4** = sum of linear product cleaved with methylamine from the relevant PCP-domain.

## Conclusions

Condensation domains are essential for non-ribosomal peptide synthesis and the identified diversity of function of these domains is rapidly increasing.^3^,^5^,^8^ Given the central role of these domains with NRPS synthesis, it is essential that we understand the selectivity and interplay of these domains in order to gain a complete overview of NRPS assembly lines and as a prequel to successful bioengineering to produce novel NRPS products. In this study, we have characterised the C-domain from the final NRPS module of GPA biosynthesis due (i) to its pivotal role in heptapeptide assembly prior to peptide cyclisation, (ii) the unusual evolutionary origins of this domain and (iii) the general lack of characterisation of C-domains acting late within NRPS assembly lines in order to address the effects of peptide structure, stereochemistry and crosslinking on peptide bond formation. Our results show that this C-domain is tolerant of changes to the amino acids contained within the peptide and that this domain is also able to accept both l- and d-configured peptides with regards to their C-terminal residue. This result serves to illustrate that the typical expectation of a C-domain to be a stereochemical gatekeeper in isolation during NRPS-mediated synthesis is not always correct and that such selectivity also clearly depends on the presence or absence of a neighbouring E-domain. The rate of acceptance of crosslinked peptides by this C-domain is significantly slower than for the corresponding linear peptides, which reinforces the role of the unique X-domain in the final NRPS module as the site of all crosslinking during GPA biosynthesis. Finally, we have been able to demonstrate that C-domain mediated peptide bond formation is closely linked to the rate of amino acid activation performed by the downstream A-domain, with a reduction in A-domain rate leading to a concomitant increase in hydrolysis of the neighbouring C-domain donor peptide substrate. This is an important result in the context of potential enzymatic redesign for such NRPS systems, as it underlines the importance of maintaining the overall rate of peptide synthesis in order to prevent unwanted peptide hydrolysis due to a loss of productive coupling of A- and C-domain activity. Overall, our results strongly suggest that the characterisation of complete NRPS modules – combining the analysis of A-domain and C-domain selectivity as well as coupled peptide bond formation – is essential if we are to understand and in future successfully redesign the function of these complex enzymatic assembly lines.

## Conflicts of interest

There are no conflicts to declare.

## Supplementary Material

Supplementary information
