# Supplementary material for: The biosynthetic implications of late-stage condensation domain selectivity during glycopeptide antibiotic biosynthesis
Source: Chem Sci. 2018 Oct 10;10(1):118–33. doi: 10.1039/c8sc03530j (PMC6333238; doi:10.1039/c8sc03530j)
Supplement: Supplementary file 1 [file SC-010-C8SC03530J-s001.pdf]

Electronic Supplementary Material (ESI) for Chemical Science.
This journal is © The Royal Society of Chemistry 2018

Supporting Information

The biosynthetic implications of late-stage condensation domain selectivity during glycopeptide antibiotic biosynthesis

Melanie Schoppet,[a,b] Madeleine Peschke,[b] Anja Kirchberg,[a] Vincent Wiebach,[d] Roderich D. Süssmuth,[d] Evi Stegmann*[e,f] and Max J. Cryle*[a,b,c]

[a] The Monash Biomedicine Discovery Institute, Department of Biochemistry and Molecular Biology, Monash University, Clayton, Victoria 3800, Australia; EMBL Australia, Monash University, Clayton, Victoria 3800, Australia.

[b] Department of Biomolecular Mechanisms, Max Planck Institute for Medical Research, Jahnstrasse 29, 69120 Heidelberg, Germany.

[c] ARC Centre of Excellence in Advanced Molecular Imaging, Monash University, Clayton, Victoria 3800, Australia.

[d] Institut für Chemie, Technische Universität Berlin, Strasse des 17. Juni 124, 10623 Berlin, Germany.

[e] Interfaculty Institute of Microbiology and Infection Medicine Tuebingen, Microbiology/Biotechnology, University of Tuebingen, Auf der Morgenstelle 28, 72076 Tuebingen, Germany.

[f] German Centre for Infection Research (DZIF), Partner Site Tuebingen, Tuebingen, Germany.

* Address correspondence to: PD Dr. Evi Stegmann (evi.stegmann@biotech.uni-tuebingen.de) and A/Prof Dr. Max Cryle (max.cryle@monash.edu)

Table of Contents

Characterisation of teicoplanin-type hexapeptide (**1**) 1
Characterisation of actinoidin-type hexapeptide (**4**) 3
List of all peptide-CoA thioesters 5
Species identified based on mass spectral analysis of balhimycin producer strains 7
Condensation domain assay using monocyclic hexapeptidyl-PCP substrate 10

Characterisation of teicoplanin-type hexapeptide (1)

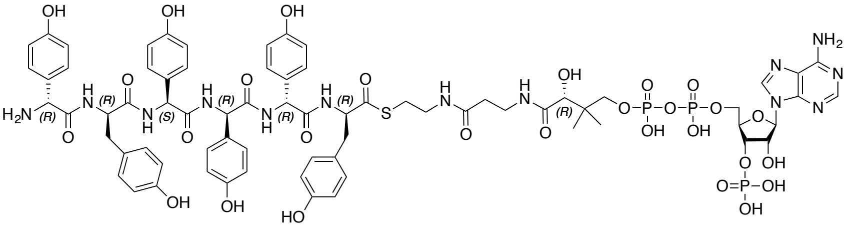

HPLC trace

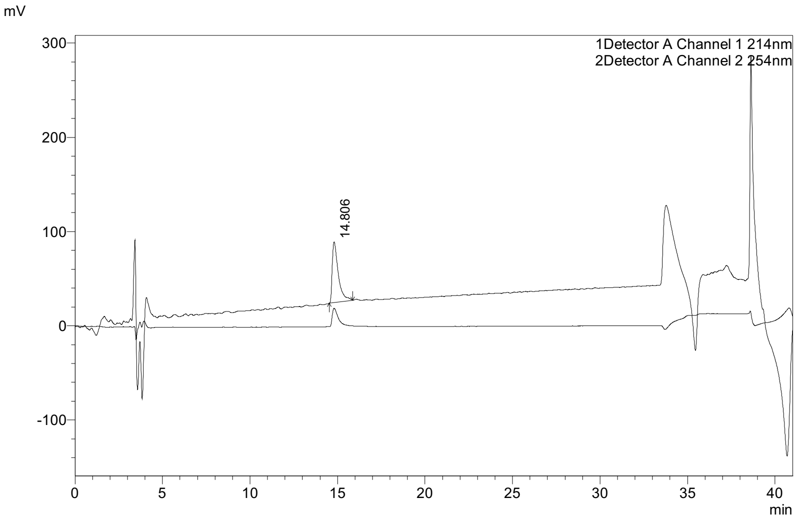

^{1}H NMR spectra

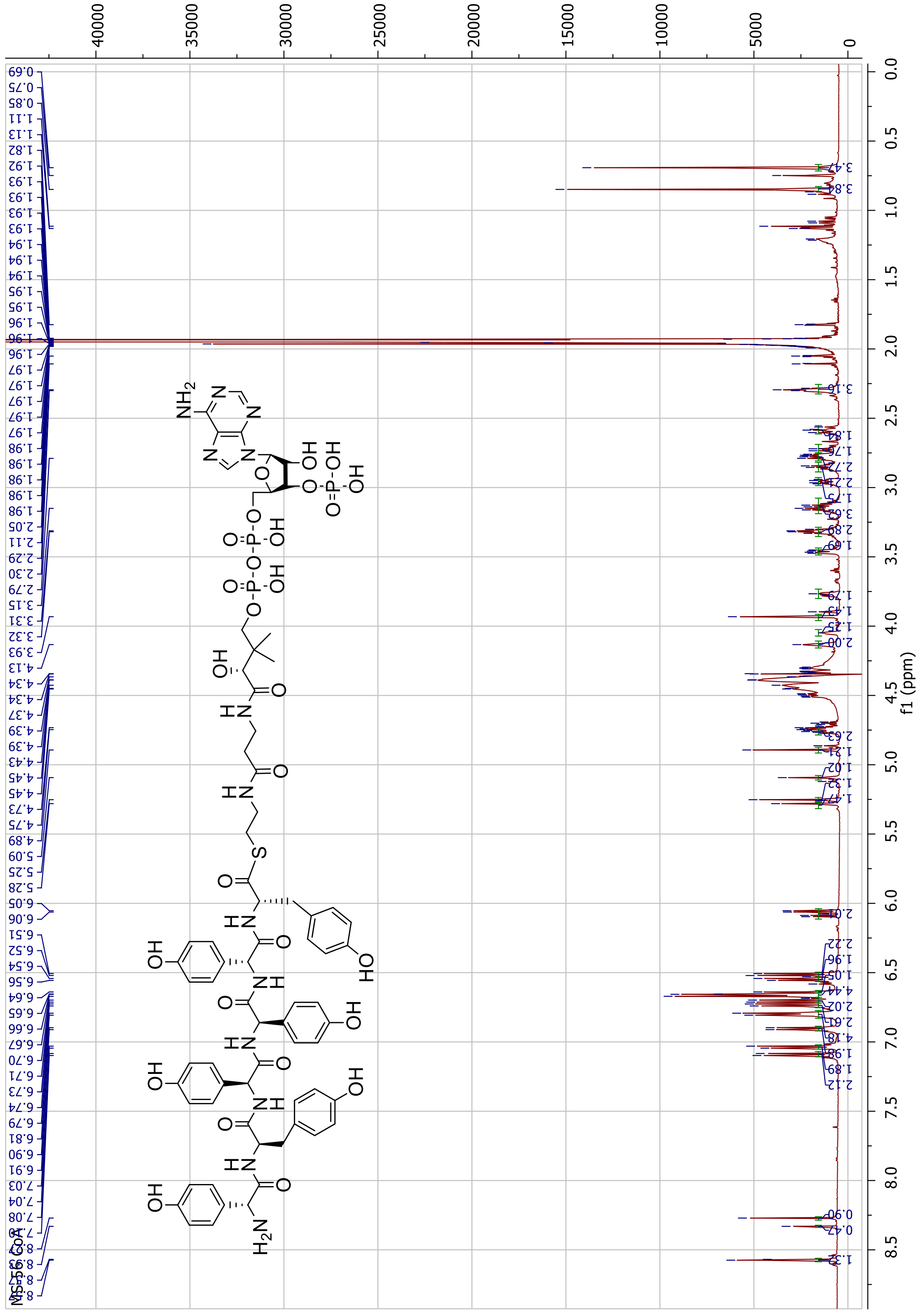

Characterisation of actinoidin-type hexapeptide (4)

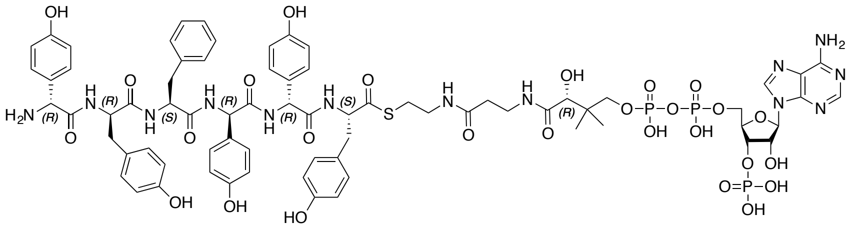

HPLC trace

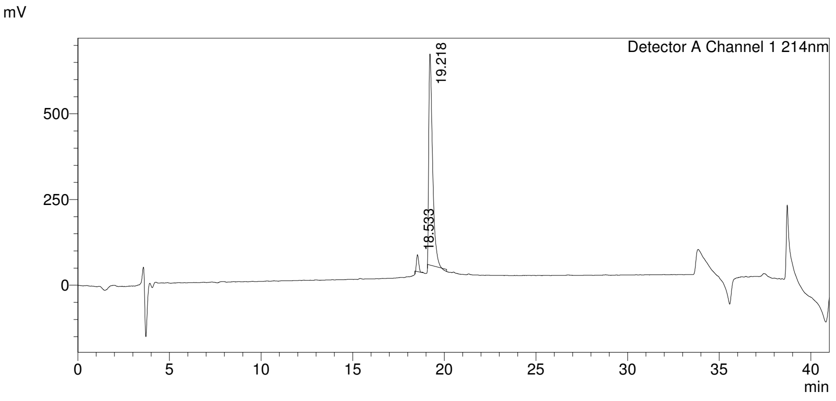

^{1}H NMR spectra

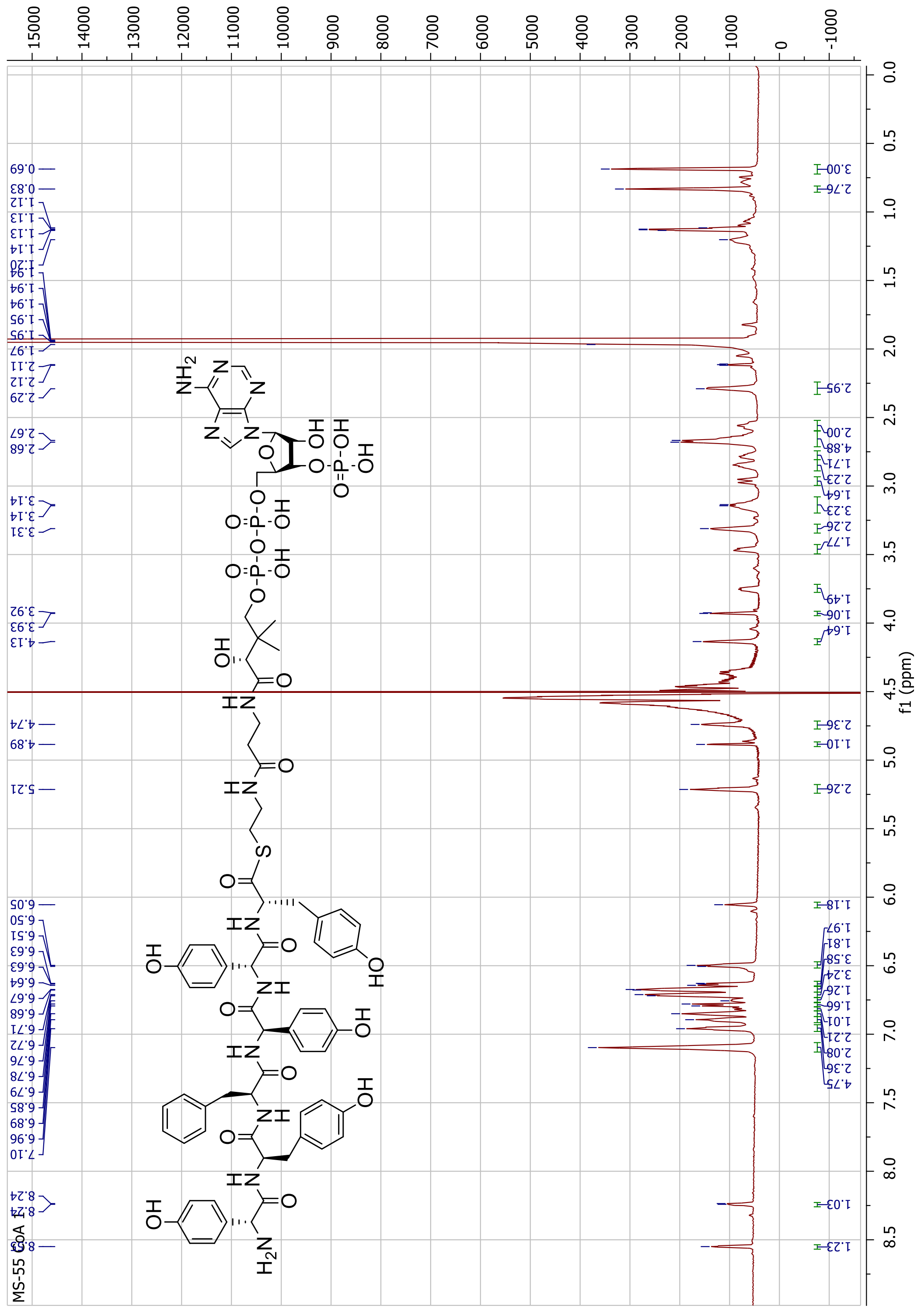

List of all peptide-CoA thioesters

Table SI1: Structure and chemical formula of all peptide-CoA thioesters synthesised in this study.

**1**<br>**Chemical Formula:**<br>$C_{71}H_{82}N_{13}O_{28}P_3S$	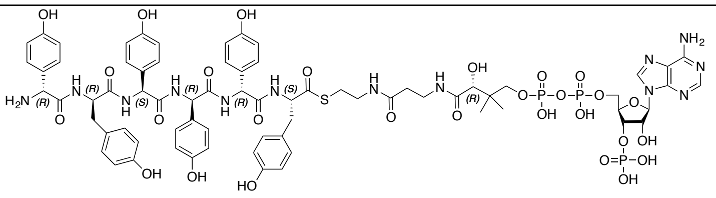
**2**<br>**Chemical Formula:**<br>$C_{71}H_{82}N_{13}O_{27}P_3S$	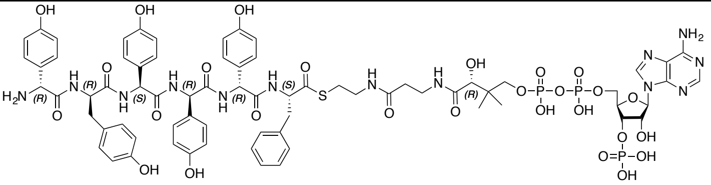
**3**<br>**Chemical Formula:**<br>$C_{72}H_{81}N_{14}O_{27}P_3S$	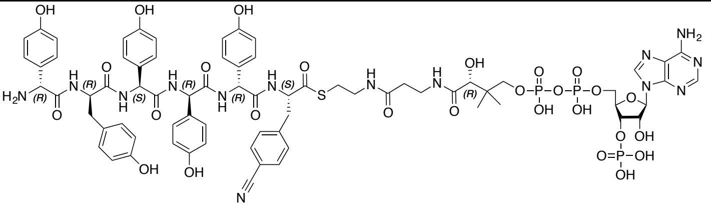
**4**<br>**Chemical Formula:**<br>$C_{72}H_{84}N_{13}O_{27}P_3S$	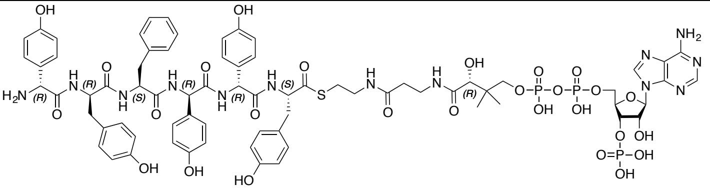
**5**<br>**Chemical Formula:**<br>$C_{71}H_{80}Cl_2N_{13}O_{28}P_3S$	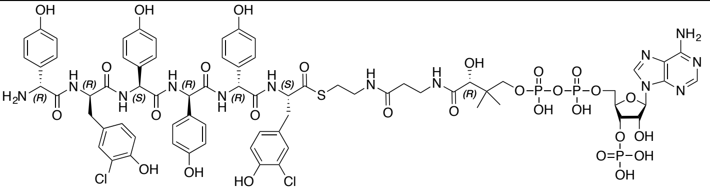
**6**<br>**Chemical Formula:**<br>$C_{72}H_{82}Cl_2N_{13}O_{27}P_3S$	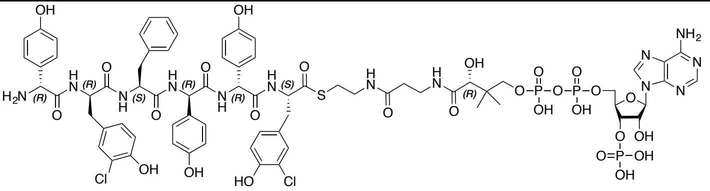
**D-1***<br>**Chemical Formula:**<br>$C_{71}H_{82}N_{13}O_{28}P_3S$	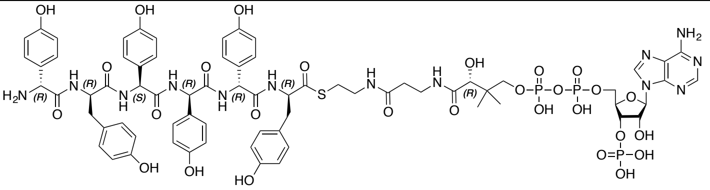

**D-4***<br>**Chemical Formula:**<br>$C_{72}H_{84}N_{13}O_{27}P_3S$	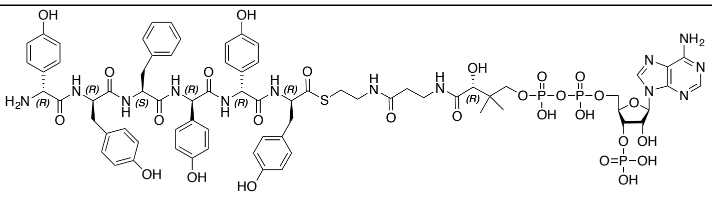
**7**<br>**Chemical Formula:**<br>$C_{62}H_{73}N_{12}O_{26}P_3S$	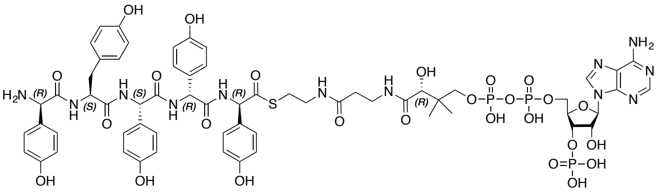
**8**<br>**Chemical Formula:**<br>$C_{63}H_{75}N_{12}O_{25}P_3S$	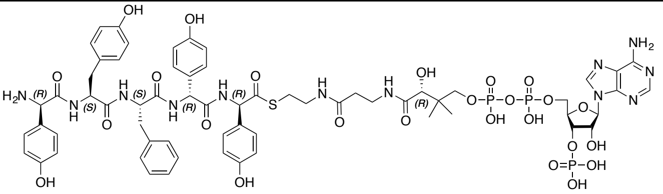
**9**<br>**Chemical Formula:**<br>$C_{63}H_{74}ClN_{12}O_{25}P_3S$	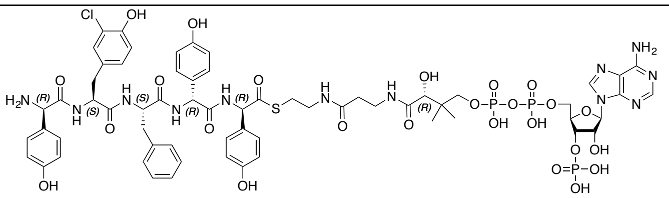

*D indicates a D-amino acid residue in position six of the peptides instead of the natural L-amino acid.

Species identified based on mass spectral analysis of balhimycin producer strains

***A. balhimycina*_ΔbpsCX**			
	Theoretical	Observed	Error (ppm)
Bal linear heptapeptide (SP-1135, **2-OH**)	1135.32132	1135.31909	1.96
Bal C-*O*-D crosslinked heptapeptide (SP-1133, $\mathbf{2}_{\mathrm{mono}}$**-OH**)	1133.30567	1133.30347	1.94
Bal linear hexapeptide (SP-970, **1-OH**)	970.27873	970.27529	3.55
Bal C-*O*-D crosslinked hexapeptide (SP-968, $\mathbf{1}_{\mathrm{mono}}$**-OH**)	968.26308	968.26044	2.73
Bal linear pentapeptide (SP-757)	757.25946	757.25913	0.44

***A. balhimycina*_ΔbpsCC**			
	Theoretical	Observed	Error (ppm)
Bal linear hexapeptide (SP-970, **1-OH**)	970.27873	970.27563	3.19
Bal C-*O*-D crosslinked hexapeptide (SP-968, $\mathbf{1}_{\mathrm{mono}}$**-OH**)	968.26308	968.26058	2.58
Bal linear pentapeptide (SP-757)	757.25946	757.25891	0.73

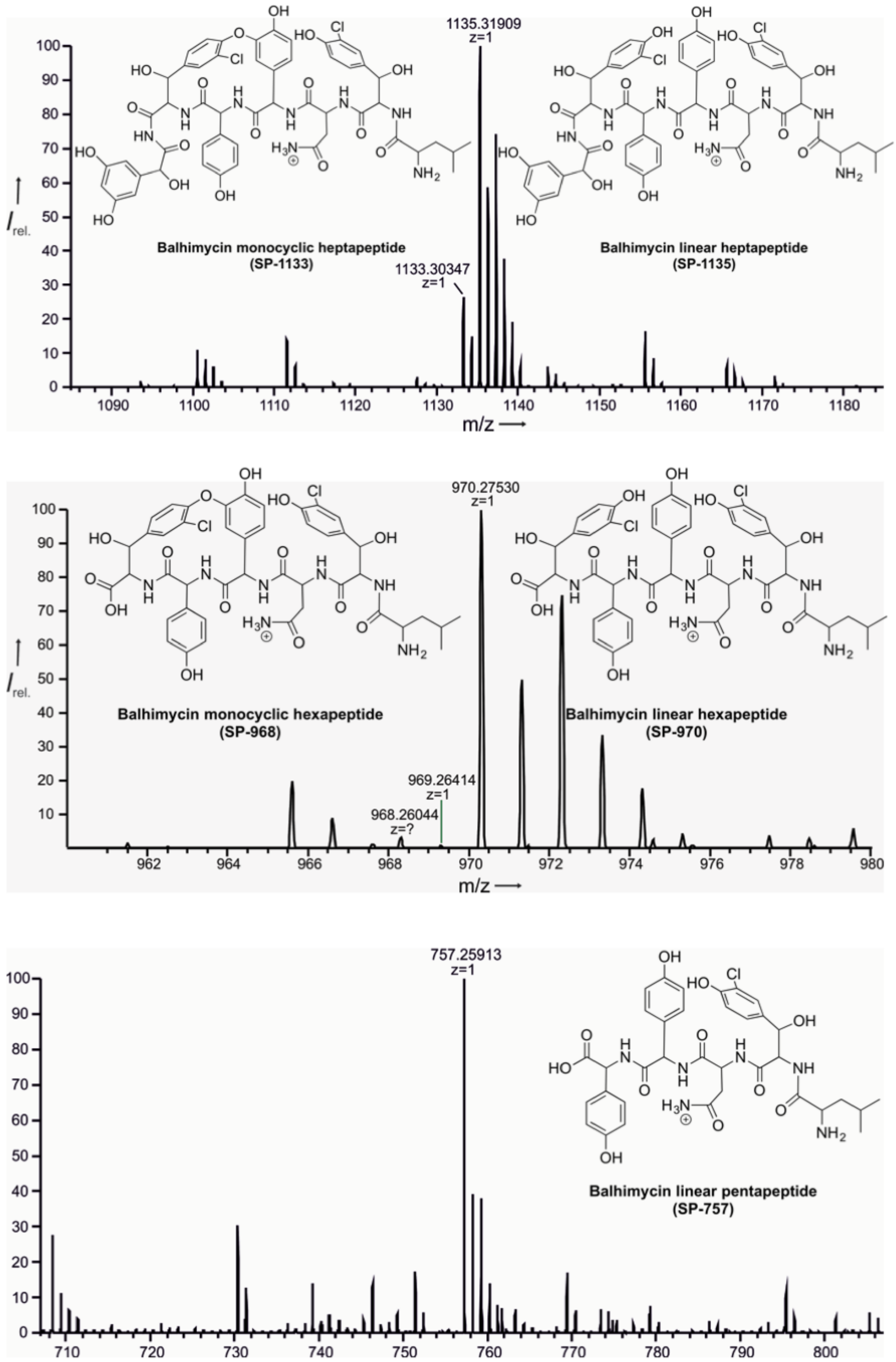

Figure SI1: Major products identified from the extract of strain ***A. balhimycina*_ΔbpsCX**. Results indicate the formation of hepta-, hexa- and pentapeptides, with monocrosslinked species identified for both hepta- and hexapeptides. All peptide species are chlorinated due to the halogenation of specific aminoacyl-PCPs that occurs during NRPS-mediated peptide synthesis.

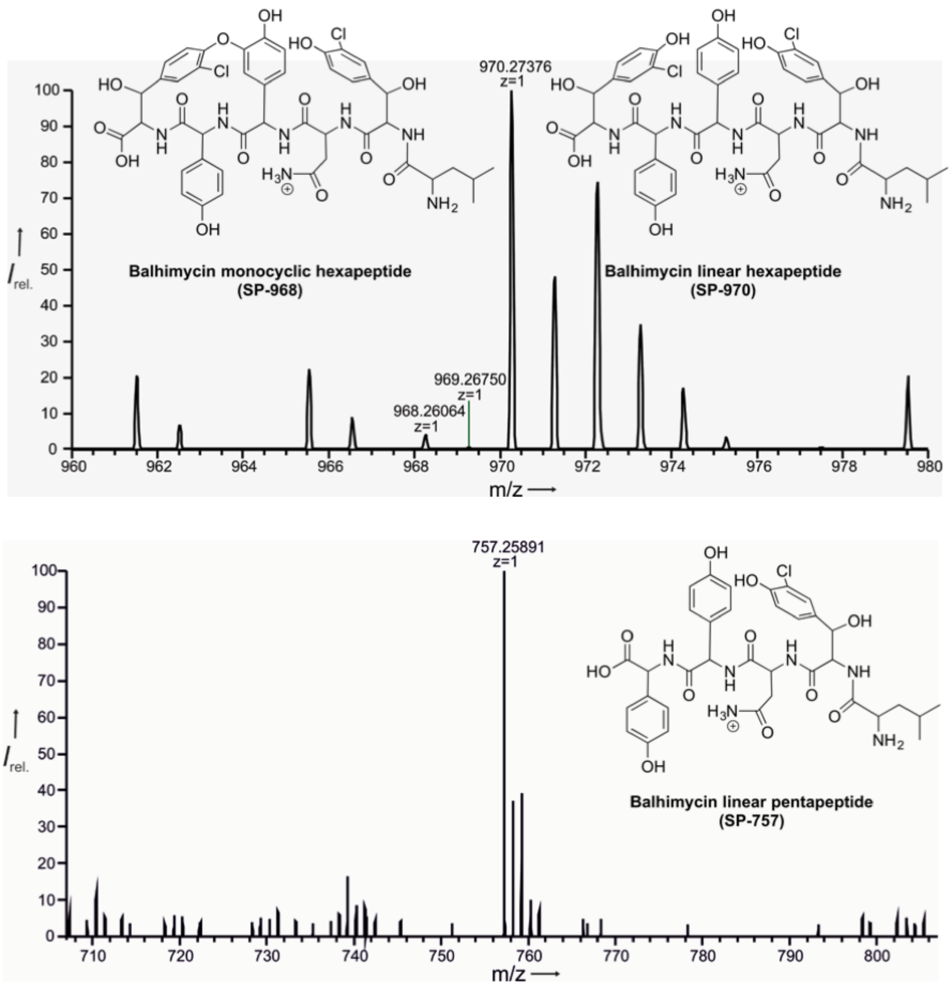

Figure SI2: Major products identified from the extract of strain ***A. balhimycina*****_ΔbpsCC**. Results indicate the formation of hexa- and pentapeptides, with monocrosslinked species identified for the hexapeptide species. All peptide species are chlorinated due to the halogenation of specific aminoacyl-PCPs that occurs during NRPS-mediated peptide synthesis.

Condensation domain assay using monocyclic hexapeptidyl-PCP substrate (Mono-4)

Hexapeptide **4** was loaded onto the stand-alone PCP_6 using Sfp as already described and enzymatic transformation with $OxyB_{bal}$ (plus competent redox system) was performed for 1 h, after which a sample was taken and analysed to assess cyclisation progress to produce **Mono-4**. Subsequently, Tcp12ΔTE_2, ATP and Dpg were added and time points were taken after 5 min, 10 min, 30 min, 60 min and 180 min (single experiments). Results indicate that peptide bond formation using the monocyclic hexapeptide (**Mono-4-PCP$_6$**) is possible, although at a significantly reduced rate over that of the linear hexapeptide.

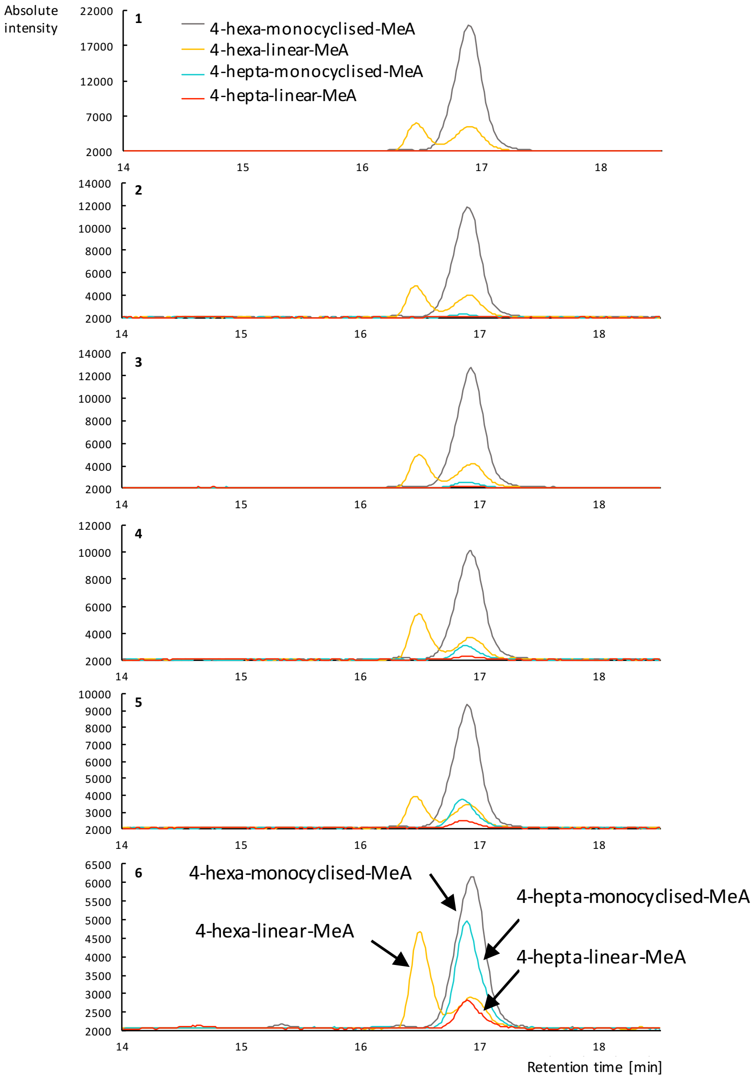

Figure SI3: Comparison of the MS traces (negative mode, SIM) after different time points. Trace 1 incubation of hexapeptide **4-PCP$_6$** with $OxyB_{van}$ for 1 h; Trace 2 addition of Tcp12ΔTE_{2l} and Dpg after 5 min; Trace 3 after 10 min; Trace 4 after 30 min; Trace 5 after 60 min; Trace 6 after 180 min.
